# Supplementary material for: Assessing machine learning for fair prediction of ADHD in school pupils using a retrospective cohort study of linked education and healthcare data
Source: BMJ Open. 2022 Dec 5;12(12):e058058. doi: 10.1136/bmjopen-2021-058058 (PMC9723859; doi:10.1136/bmjopen-2021-058058)

Table S2: Educational Features

	Feature name	Feature Description
Demographic features	Gender (Male)	Dummy variable indicating male gender.
	Age	Age in years and months at the start of KS1.
	Summer birth	Variable indicating whether the child was born in summer months of school term (1st April- 31st August). These are the youngest children in an academic cohort.
	Ethnicity	Dummy variables indicating ethnic background. We included 6 separate ethnicity dummy variables within the model: Asian, Black, Chinese, White, Mixed, Other.
	Deprivation Indicator (IDACI)	The Income Deprivation Affecting Children Index (IDACI) scores are an indicator of deprivation. The IDACI measures the proportion of all children aged 0 to 15 living in income deprived families. It is a subset of the Income Deprivation Domain which measures the proportion of the population in an area experiencing deprivation relating to low income.
	Academic Year	This feature describes the academic year a child started KS1 (for example 2011).
Additional need indicators	eFSM	Eligibility for Free School Meals is a statutory benefit available to school aged children in England from less affluent backgrounds. eFSM is a commonly used proxy variable for lower socio-economic status.
	Mobility (KS1)	Variable indicating whether a child has moved schools within KS1.
	Mobility (ever)	Variable indicating whether a child has moved schools ever (i.e. during KS1 or the EYFSP).
	Child Looked After Status	Child looked after status indicates that a child has been in the care of their local authority for more than 24 hours.
	English first language	English as a first language refers to whether the child's native language is English or other.
Special Educational Need indicators	No SEN	Dummy variable indicating that the child had no Special Educational Needs.
	SEN School Action	Dummy variable indicating that a child has a school action plan to support their SEN. This might include adapted teaching methods and materials, or in some cases additional adult support. This plan is created by the school.
	SEN School Action+	Dummy variable indicating that a child has had a school action plan to support their SEN, however this has not helped the child make adequate progress. At this point school action plus is required. This involves schools consulting and collaborating with appropriate external support services (for example, a speech and language therapist).
	SEN Statement of needs	Dummy variable indicating that child has a SEN statement of needs. A SEN Statement is a plan set by the Education Authority detailing a child's needs and required support. This is typically made if a child needs support beyond the scope of the school (school action/ school action plus).
	SEN: Autism spectrum disorders	Autism spectrum disorder is a specific SEN. It is a diagnosable disorder that affects social interaction, interests and behaviour.
	SEN: specific learning difficulty	Children with specific learning difficulties, typically struggle with one specific aspect of learning but are able in other areas. For

		example, they may find reading particularly challenging (but cope well with numbers and letters).
	SEN: moderate learning difficulty	Learning difficulties are formally diagnosed by a clinician. The SEN: moderate learning difficulty code indicates a child's ability to understand and communicate information is moderately impaired.
	SEN: severe learning difficulty	Learning difficulties are formally diagnosed by a clinician. The SEN: severe learning difficulty code indicates a child's ability to understand and communicate information is severely impaired.
	SEN: behavioural, emotional and social difficulties	SEN: Behavioural, social and emotional difficulties is a broad term referring to children who's behaviour poses a barrier to learning. This can be vary in terms of presentation, children may be withdrawn or isolated, disruptive, lack concentration, immature social skills, or other challenging behaviours.
	SEN: speech, language & communication needs	This variable highlights those who find it difficult to listen, understand or communicate with others that adversely affects educational performance.
	SEN: hearing impairment	This variable indicates a child who has either a permanent or fluctuating hearing impairment that adversely affects educational performance.
	SEN: vision impairment	This variable indicates a child who has either a permanent or fluctuating visual impairment that adversely affects educational performance.
	SEN: multi-sensory impairment	This variable indicates a child who has either a permanent or fluctuating multi-sensory impairment (e.g. hearing and vison) that adversely affects educational performance.
	SEN: physical	This variable indicates a child who has either a permanent or fluctuating physical impairment that adversely affects educational performance.
	SEN: other	This variable indicates a child who has a special educational need which is adversely affecting education performance, but not captured in the categories above.
	Mainstream education indicator	Dummy variable indicating that a child is in mainstream education (as opposed to alternative provision).
School attendance and exclusion features	Attendance (%)	The percentage of time within the school year that a child was registered as attending school.
	Exclusion (temporary)	A fixed period exclusion where a child is temporarily moved from school (a maximum of 45 days within a school year).
	Exclusion (permanent)	Permanent exclusion indicating a child has been expelled from school.
School attainment features	EYFSP knowledge of the world	EYFSP sub scale: assesses a child's ability to make sense of their physical world and community on a scale of 1-9 (with 9 being most proficient). This is not a standardised assessment, however it is conducted by a trained professional (Early Years Practitioner) and moderated by the local authority.
	EYFSP problem solving, reasoning and numeracy	EYFSP sub scale: assesses a child's ability to problem solve, reason and perform basic counting and calculations on a scale of 1-9 (with 9 being most proficient). This is not a standardised assessment, however it is conducted by a trained professional (Early Years Practitioner) and moderated by the local authority.
	EYFSP personal, social and emotional development	EYFSP sub scale: assesses a child's personal, social and emotional development on a scale of 1-9 (with 9 being most proficient). This is not a standardised assessment, however it is conducted by a

Ter-Minassian L, *et al. BMJ Open* 2022; 12:e058058. doi: 10.1136/bmjopen-2021-058058

	trained professional (Early Years Practitioner) and moderated by the local authority.
EYFSP communication, language and literacy	EYFSP sub scale: assesses a child's communication, language and literacy performance on a scale of 1-9 (with 9 being most proficient). This is not a standardised assessment, however it is conducted by a trained professional (Early Years Practitioner) and moderated by the local authority.
EYFSP physical development	EYFSP sub scale: assesses a child's physical development on a scale of 1-9 (with 9 being most proficient). This is not a standardised assessment, however it is conducted by a trained professional (Early Years Practitioner) and moderated by the local authority.
EYFSP creative development	EYFSP sub scale: assesses a child's creative development on a scale of 1-9 (with 9 being most proficient). This is not a standardised assessment, however it is conducted by a trained professional (Early Years Practitioner) and moderated by the local authority.
KS1 Maths	National Standardised Test: Mathematics assessment score.
KS1 Writing	National Standardised Test: Writing assessment score.
KS1 Reading	National Standardised Test: Reading assessment score.
KS1 Science	National Standardised Test: Science assessment score.

Note: Educational features are drawn from the National Pupil Database (NPD). The NPD is a longitudinal database which holds individual-level records for all children and young people enrolled in state school, or taking national exams, and are currently residing in England. The database is large, with over 15 million records added since it's introduction (2002). The NPD holds information on a child's attainment, attendance, school context, and level of need (in addition to demographic variables). Data is provided on a quarterly basis by all state funded primary and secondary schools across England, via a dedicated submission system to the government's Department for Education.

Table S3: Educational feature time points

		EYFSP	KS1
Demographic features	Gender (Male)	✓	✓
	Age	✓	✓
	Summer Birth	✓	✓
	Ethnicity	✓	✓
	Deprivation Indicator (IDACI)	✓	✓
	Academic Year		✓
Additional need indicators	eFSM	✓	✓
	Mobility (KS1)		✓
	Mobility (ever)		✓
	Child Looked After Status	✓	✓
	English first language	✓	✓
Special Educational Need indicators	No SEN	✓	✓
	SEN Statement	✓	✓
	SEN School Action	✓	✓
	SEN School Action+	✓	✓
	SEN: Autism spectrum disorders	✓	✓
	SEN: specific learning difficulty	✓	✓
	SEN: moderate learning difficulty	✓	✓
	SEN: severe learning difficulties	✓	✓
	SEN: behavioural, emotional and social difficulty	✓	✓
	SEN: speech, language & communication needs	✓	✓
	SEN: hearing impairment	✓	✓
	SEN: vision impairment	✓	✓
	SEN: multi-sensory impairment	✓	✓
	SEN: physical	✓	✓
	SEN: other	✓	✓
	Mainstream education indicator		✓
School attendance and exclusion features	Attendance (%)	✓	✓
	Excluded (temporary)		✓
	Excluded (permanent)		✓
School attainment features	EYFSP knowledge of the world	✓	
	EYFSP problem solving, reasoning and numeracy	✓	
	EYFSP personal, social and emotional development	✓	
	EYFSP communication, language and literacy	✓	
	EYFSP physical development	✓	
	EYFSP creative development	✓	
	KS1 Maths		✓
	KS1 Writing		✓
	KS1 Reading		✓
	KS1 Science		✓

Table S4: Tuned hyperparameters

Logistic Regression	solver
Random Forest	n_estimators, max_depth, max_features, bootstrap, criterion, min_samples_split
SVM	C, cache_size, degree, gamma, kernel, max_iter, tol=0.001
Gaussian Bayes	No tuning
MLP	weight_decay, batch_size, dropout_rate, number of hidden layers

Ter-Minassian L, *et al. BMJ Open* 2022; 12:e058058. doi: 10.1136/bmjopen-2021-058058

Table S5: AUC comparison between ROC and PRC analyses for population and clinical classification of ADHD.

	**Population cohort**		**Clinical cohort**	
**Classification Model**	ROC[c] AUC[d]	PRC[e] AUC[d]	ROC[c] AUC[d]	PRC[e] AUC[d]
**LR[a]**	0.900	0.101	0.694	0.244
**RF[b]**	0.86	0.070	0.688	0.257
**Random**	0.50	0.029	0.50	0.21

[a] Logistic Regression (LR), [b]Random Forest (RF), [c] Receiver Operating Characteristic curve (ROC)
[d] Area Under Curve (AUC) [e] Precision-recall curve

Ter-Minassian L, *et al. BMJ Open* 2022; 12:e058058. doi: 10.1136/bmjopen-2021-058058

Table S6: Pupil characteristics and feature contribution (unweighted) for ADHD prediction in the general population sample.

Characteristics	Logistic Regression (***B*** co-efficient)	Random Forest (feature importance)
Male Gender	0.608463661	0.141773129
Summer Born (May-August)	0.109707469	0.000807359
White Ethnic group	0.201065669	0.006387057
Black Ethnic group	-0.098073916	0.000345206
Asian Ethnic Group	-0.15535868	0.011452877
Chinese Ethnic group	-0.469369952	0
Mixed Ethnic group	0.165278809	0.001506941
Other Ethnic Group	-0.065271609	0.002849315
English as first language	0.696034466	0.031602634
EYFSP: FSM eligible	0.110110336	0.001856049
EYFSP: Looked After Child	-0.058157067	0
EYFSP: IDACI score	-0.245109352	0.012435495
EYFSP: Communication, Language and Literacy	0.180721245	0.01633496
EYFSP: Knowledge of the World z score	0.436711733	0.011483452
EYFSP: Person, social, emotion z score	-0.84127724	0.047292232
EYFSP: Physical Development	-0.027708196	0.007938476
EYFSP: Creative Development	0.123449383	0.01211572
EYFSP: Problem solving, reasoning and numeracy	0.224475588	0.019006476
school mobility (ever)	-0.066303394	0.001939589
EYFSP: attendance (%)	-0.982714854	0.050421392
EYSFSP local school	-0.077583539	0.0017516
EYSFSP school type	-0.074490398	0.003640833
EYFSP: No SEN	-0.105148801	0.093914996
EYFSP: SEN School Action	0.048150906	0.008735734
EYFSP: SEN School Action Plus	0.122221511	0.030373947
EYFSP: SEN Statement	-0.044818697	0
EYFSP: SEN speech, language & communication needs	-0.113501845	0.001918025
EYFSP: SEN Autism	-0.027233555	0
EYFSP: SEN behavioural, emotional and social difficulty	-0.087858945	0.005794567
EYFSP: SEN Hearing	0.002234986	0.002198098
EYFSP: SEN Moderate learning difficulty	-0.002195983	0.000564267
EYFSP: SEN Other	0.046557778	0.00206679
EYFSP: SEN Physical	-0.043438673	0.003612997
EYFSP: SEN Specific learning difficulty	0.008096732	0
EYFSP: SEN Severe learning difficulty	-0.087701227	0.000828542
EYFSP: SEN Vision	-0.014857814	0
EYFSP: SEN Multi-sensory impairment	-0.137831643	0
KS1: Age at start	0.100899579	0.018690101
KS1 : Academic Year (e.g. 2008/9)	-0.046794005	0.022486708
KS1: mainstream school	-0.064770905	0.002768431

KS1: FSM eligible	0.113473204	0.001361182
KS1: IDACI score	0.17501001	0.006385568
KS1: Looked After Child	0.054056081	0.001767296
KS1: Maths score	-0.164521477	0.032731843
KS1: Writing point score	-0.761201837	0.10916803
KS1: Read Score	0.001961117	0.035857909
KS1: Science score	-0.028166159	0.010063037
KS1 school mobility	0.054731598	0.001614064
KS1: attendance (%)	0.957564403	0.059408817
KS1: Excluded	0.125124512	0.004965946
KS1: Exclusion	0.024925737	0.005752298
KS1: Not SEN	-0.198059867	0.08701934
KS1: SEN School Action	0.063043447	0.003333059
KS1: SEN status: school action plus	0.175904692	0.020499615
KS1: SEN: speech, language & communication needs	-0.030230103	0.000800672
KS1: SEN Autism	0.147169063	0.002138406
KS1: SEN behavioural, emotional and social difficulty	-0.087858945	0.00538704
KS1: SEN Hearing	0.002234986	0.001026292
KS1: SEN Moderate learning difficulty	-0.002195983	0.000752458
KS1: SEN Multi-sensory impairment	-0.137831643	0
KS1: SEN Other	0.046557778	0.007853115
KS1: SEN Physical	-0.043438673	0.004452396
KS1: SEN Specific learning difficulty	0.008096732	0.000377725
KS1: SEN Severe Learning Difficulty	0.043312749	0
KS1: SEN Statement	0.09702835	0
KS1: SEN Vision	-0.014857814	0

Ter-Minassian L, *et al. BMJ Open* 2022; 12:e058058. doi: 10.1136/bmjopen-2021-058058

Figure S1: Pre-processing fairness pipeline

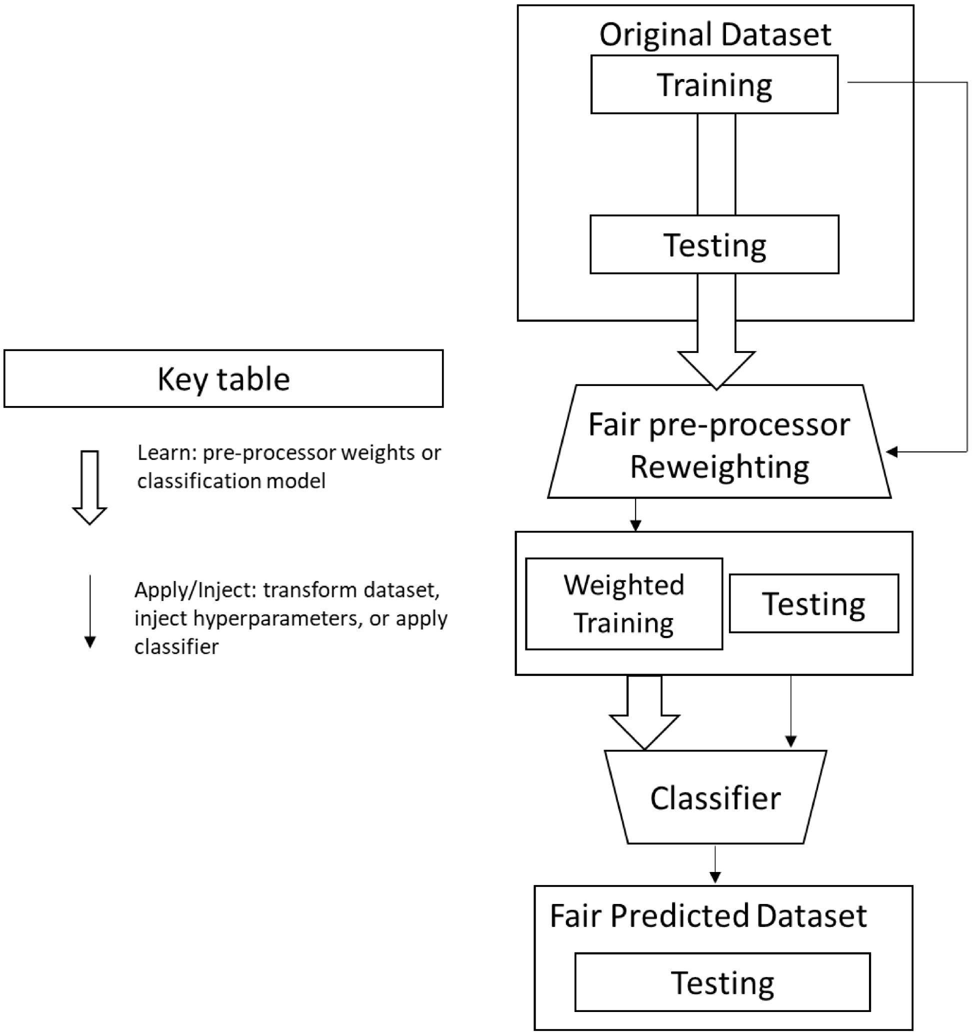

Figure S2: Precision-Recall curves comparing Logistic Regression(LR), Random Forest (RF) and a Random classifier for ADHD diagnosis within a population cohort

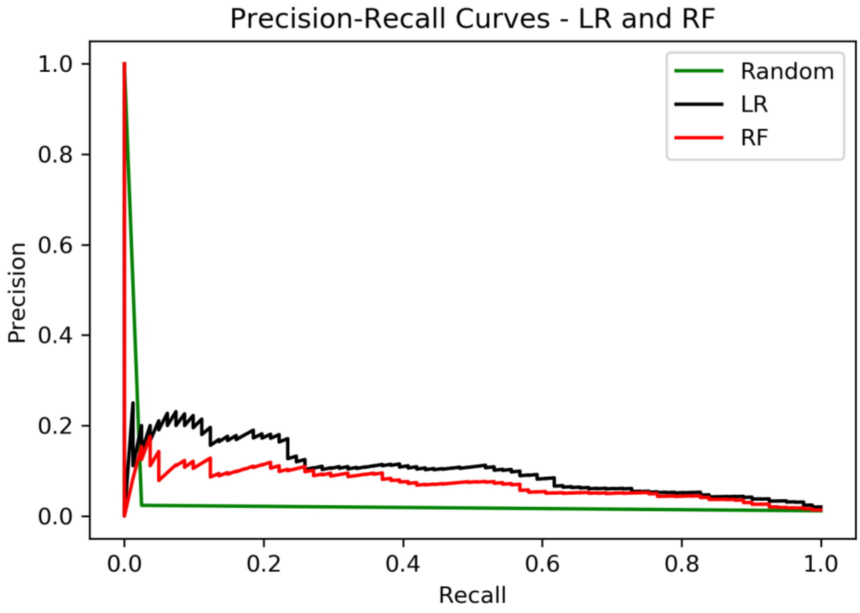

Figure S3: Precision-Recall curves comparing Logistic Regression(LR), Random Forest(RF) and a Random classifier for ADHD diagnosis within a clinical cohort

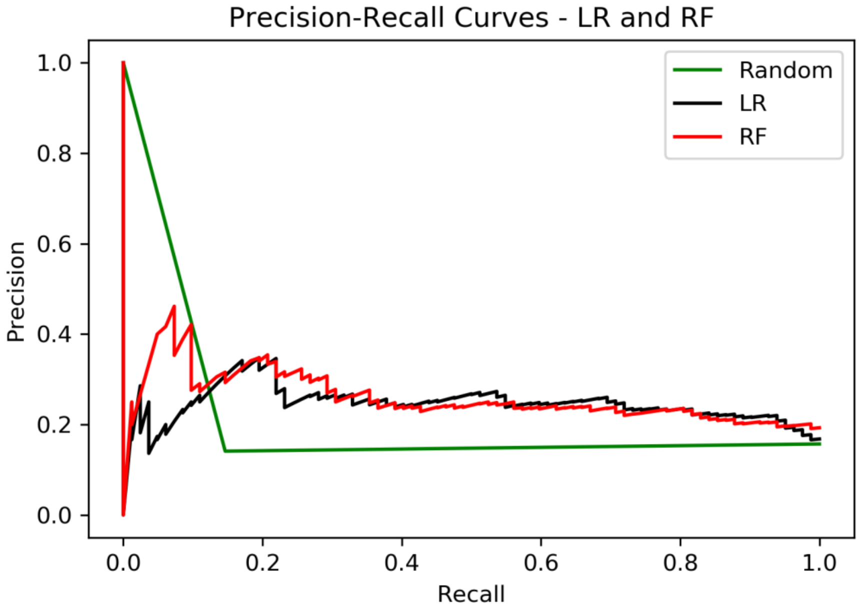

Supplement: Supplementary data [file bmjopen-2021-058058supp002.pdf]
