# Supplementary material for: Assessing machine learning for fair prediction of ADHD in school pupils using a retrospective cohort study of linked education and healthcare data
Source: BMJ Open. 2022 Dec 5;12(12):e058058. doi: 10.1136/bmjopen-2021-058058 (PMC9723859; doi:10.1136/bmjopen-2021-058058)
Supplement: Author's manuscript [file bmjopen-2021-058058.draft_revisions.pdf]

BMJ Open is committed to open peer review. As part of this commitment we make the peer review history of every article we publish publicly available.

When an article is published we post the peer reviewers' comments and the authors' responses online. We also post the versions of the paper that were used during peer review. These are the versions that the peer review comments apply to.

The versions of the paper that follow are the versions that were submitted during the peer review process. They are not the versions of record or the final published versions. They should not be cited or distributed as the published version of this manuscript.

BMJ Open is an open access journal and the full, final, typeset and author-corrected version of record of the manuscript is available on our site with no access controls, subscription charges or pay-per-view fees (http://bmjopen.bmj.com).

If you have any questions on BMJ Open's open peer review process please email info.bmjopen@bmj.com

BMJ Open

Assessing machine learning for fair prediction of ADHD in school pupils using an innovative linkage between education and healthcare data

Journal:	*BMJ Open*
Manuscript ID	bmjopen-2021-058058
Article Type:	Original research
Date Submitted by the Author:	06-Oct-2021
Complete List of Authors:	Ter-Minassian, Lucile; King's College London, Department of Psychological Medicine<br>Viani, Natalia; King's College London, Department of Psychological Medicine<br>Wickersham, Alice; King's College London, Institute of Psychiatry, Psychology and Neuroscience<br>Cross, Lauren; King's College London, Department of Psychological Medicine<br>Stewart, Robert; King's College London, Institute of Psychiatry<br>Velupillai, Sumithra; Kings College London, Institute of Psychiatry, Psychology and Neuroscience<br>Downs, Johnny; South London and Maudsley NHS Foundation Trust, Department of Child and Adolescent Psychiatry
Keywords:	Child & adolescent psychiatry < PSYCHIATRY, EPIDEMIOLOGY, MENTAL HEALTH

SCHOLARONE™
Manuscripts

I, the Submitting Author has the right to grant and does grant on behalf of all authors of the Work (as defined in the below author licence), an exclusive licence and/or a non-exclusive licence for contributions from authors who are: i) UK Crown employees; ii) where BMJ has agreed a CC-BY licence shall apply, and/or iii) in accordance with the terms applicable for US Federal Government officers or employees acting as part of their official duties; on a worldwide, perpetual, irrevocable, royalty-free basis to BMJ Publishing Group Ltd ("BMJ") its licensees and where the relevant Journal is co-owned by BMJ to the co-owners of the Journal, to publish the Work in this journal and any other BMJ products and to exploit all rights, as set out in our licence.

The Submitting Author accepts and understands that any supply made under these terms is made by BMJ to the Submitting Author unless you are acting as an employee on behalf of your employer or a postgraduate student of an affiliated institution which is paying any applicable article publishing charge ("APC") for Open Access articles. Where the Submitting Author wishes to make the Work available on an Open Access basis (and intends to pay the relevant APC), the terms of reuse of such Open Access shall be governed by a Creative Commons licence – details of these licences and which Creative Commons licence will apply to this Work are set out in our licence referred to above.

Other than as permitted in any relevant BMJ Author's Self Archiving Policies, I confirm this Work has not been accepted for publication elsewhere, is not being considered for publication elsewhere and does not duplicate material already published. I confirm all authors consent to publication of this Work and authorise the granting of this licence.

For peer review only

Title

Assessing machine learning for fair prediction of ADHD in school pupils using an innovative linkage between education and healthcare data

Author names and affiliations

Ms Lucile Ter-Minassian, MSc

Department of Psychological Medicine, Institute of Psychiatry, Psychology & Neuroscience, King's College London, London, UK.

Dr Natalia Viani*, PhD

Department of Psychological Medicine, Institute of Psychiatry, Psychology & Neuroscience, King's College London, London, UK.

Ms Alice Wickersham*, MSc

Department of Psychological Medicine, Institute of Psychiatry, Psychology & Neuroscience, King's College London, London, UK.

Ms Lauren Cross*, MSc

Department of Psychological Medicine, Institute of Psychiatry, Psychology & Neuroscience, King's College London, London, UK.

Prof Robert Stewart, MD

Department of Psychological Medicine, Institute of Psychiatry, Psychology & Neuroscience, King's College London, London, UK.

South London and Maudsley NHS Foundation Trust, London, UK

Dr Sumithra Velupillai, PhD

Department of Psychological Medicine, Institute of Psychiatry, Psychology & Neuroscience, King's College London, London, UK.

For peer review only

Dr Johnny Downs, PhD

Department of Child and Adolescent Psychiatry, King's College London, Institute of Psychiatry, Psychology & Neuroscience London, UK.

South London and Maudsley NHS Foundation Trust, London, UK

* Authors contributed equally

Corresponding author

Johnny Downs, Department of Child and Adolescent Psychiatry, Institute of Psychiatry, Psychology & Neuroscience, King's College London, Box PO84, 16 De Crespigny Park, London SE5 8AF, UK. johnny.downs@kcl.ac.uk

Word count

3,142

For peer review only

ABSTRACT

Objectives: Attention Deficit Hyperactivity Disorder (ADHD) is a prevalent childhood disorder, but often goes unrecognised and untreated. To improve access to services, accurate predictions of populations at high risk for ADHD are needed for effective resource allocation. Using a unique linked health and education data resource, we examined how machine learning (ML) approaches can predict risk of ADHD.

Design: Retrospective population cohort study.

Setting: South London (2007-2013).

Participants: n=56,258 pupils with linked education and health data.

Primary outcome measures: Using area under the curve (AUC), we compared the predictive accuracy of four ML models and one Neural Network for ADHD diagnosis. Ethnic group and language biases were weighted using a fair pre-processing algorithm.

Results: Random Forest and Logistic Regression prediction models provided the highest predictive accuracy for ADHD in population samples (AUC 0.86, 0.86 respectively) and clinical samples (AUC 0.72, 0.70). Precision-recall curve analyses were less favourable. Sociodemographic biases were effectively reduced by a fair pre-processing algorithm without loss of accuracy.

Conclusions: ML approaches using linked routinely collected education and health data offer accurate, low cost, and scalable prediction models of ADHD. These approaches could help identify areas of need and inform resource allocation. Introducing 'fairness weighting' attenuates some sociodemographic biases which would otherwise underestimate ADHD risk within minority groups.

Key words: Attention Deficit Disorder with Hyperactivity; Child Psychiatry; Machine Learning; Neural Networks; Algorithms

For peer review only

For peer review only - http://bmjopen.bmj.com/site/about/guidelines.xhtml

Strengths and limitations of this study

- Using a large linkage between routinely collected health and school data, machine learning approaches can be used to predict Attention Deficit Hyperactivity Disorder (ADHD).
- This demonstrates that prediction models can be scaled to large populations for effective resource allocation and service improvement.
- These prediction models can be weighted to account for ethnic group and language biases.
- Further validation of these models is needed to establish the stability and generalisability of these models.
- The potential impact of imbalances in the dataset on modelling, particularly the small number of ADHD cases, needs further investigation.

For peer review only

INTRODUCTION

Attention Deficit Hyperactivity Disorder (ADHD) is a neurodevelopmental disorder characterised by a persistent pattern of inattention, hyperactivity and impulsive behaviour.[1] It affects an estimated 5.3% of the global population, and although symptoms emerge in pre-school, ADHD tends to lead to clinical levels of impairment during the first few years of statutory education.[2] ADHD can lead to poor outcomes across social, educational and health domains.[3, 4] It can be treated,[5] but substantial diagnostic delays preclude effective early intervention.[6] It is therefore vital that adequate resources are made available for timely ADHD detection, diagnosis and intervention.

To effectively allocate adequate resources for ADHD, commissioners need accurate information on the level of need in schools, services and geographic regions. At the school-level, mental health screening questionnaires administered by teachers can be used to identify pupils at risk of ADHD, which in could turn provide an estimate of ADHD prevalence for that school.[7] However, this approach is resource-intensive, and cannot practically be scaled up to larger populations to estimate burden of need in a wider geographic area.[8] Machine learning techniques may be able to predict ADHD diagnosis at the individual-level, but existing approaches again use data sources which cannot be practically scaled up to larger populations, such as Magnetic Resonance Imaging and electroencephalography data.[9-12] Such

For peer review only - http://bmjopen.bmj.com/site/about/guidelines.xhtml

models might also be prone to systematic biases[13]: ADHD is under-diagnosed among ethnic minority groups, who are therefore unlikely to be adequately represented in the data on which such models are built, unless efforts are made to adjust for such biases.[14]

Therefore, there remains a need for accurate and unbiased ADHD prediction methods which are scalable to large populations or geographic regions. Administrative pupil-level data are already routinely collected and curated by most schools, and are available for whole populations.[15] If machine learning approaches can be effectively applied to these data to build validated and unbiased models predicting childhood ADHD, they could be used to estimate burden of need and guide resource allocation at area-, school- and service-levels. In this study, we applied machine learning approaches to a large, linked administrative school and clinical dataset to: (1) classify pupils in the population with a high likelihood of clinical ADHD, (2) differentiate pupils with ADHD from pupils with other clinical disorders, (3) explore the contribution of demographic, socioeconomic, and education features to an ADHD diagnosis, and (4) evaluate a potential method to reduce sociodemographic biases.

METHODS

Study design

This was a retrospective cohort study, using an existing data linkage between the National Pupil Database (NPD) and South London and Maudsley NHS Foundation Trust Child and Adolescent Mental Health Services (SLaM CAMHS) (Table S1).[16, 17]

SLaM is one of Europe's largest providers of mental healthcare, serving the London boroughs of Croydon, Lambeth, Lewisham and Southwark. SLaM is the monopoly provider of local CAMHS services, including ADHD diagnostic services.[16] Pseudonymised individual-level clinical data for these services are accessible for research via the Clinical Record Interactive Search (CRIS).

CRIS data for SLaM CAMHS services have been linked at the individual-level to the NPD. The NPD is a longitudinal database of pupil- and school-level data.[15] The NPD pupil census is collected annually and contains a snapshot of pupils attending state-maintained schools in

For peer review only - http://bmjopen.bmj.com/site/about/guidelines.xhtml

England, while NPD attainment datasets hold data for all statutory assessments that pupils complete during primary and secondary education.

Ethics approval

The CRIS data resource received ethical approval as an anonymised data set for secondary analyses from Oxfordshire REC C (Ref: 08/H0606/71+5) and NHS Health Research Authority Confidentiality Advisory Group, reference: CAG 9-08(a)/2014. At the time of submission, JD had full ongoing access to the data.

Patient and Public Involvement

The health and education data linkage used in this study was developed in consultation with several clinical, patient and caregiver groups via the SLaM Biomedical Research Centre (BRC) patient engagement programme.[17] A lay summary of the data linkage can be viewed at https://www.maudsleybrc.nihr.ac.uk/facilities/clinical-record-interactive-search-cris/cris-data-linkages/.

Participants

The analysed sample comprised children aged between 6 and 7 years, who were enrolled in mainstream state educational services (not specialist or alternative educational facilities), and who had education and attainment characteristics as captured at the Early Years Foundation Stage (EYFSP; typically aged 4-5 years) and at Key Stage 1 (KS1; typically aged 6-7 years). Pupils were included if they were resident of the SLaM catchment area from 1st September 2007 to 31st September 2013. A resulting n=57,149 pupils were eligible for inclusion.

Main outcome

Individual-level diagnosis of ADHD was measured from both structured and unstructured diagnosis fields in CRIS between September 2007 and August 2013. In structured fields, ADHD diagnosis was defined as International Classification of Diseases 10th Revision (ICD-10) codes F90.0, F90.1, F90.8, or F90.9 (Hyperkinetic, other Hyperkinetic Disorders, Attention Deficit Disorder, Hyperkinetic Conduct Disorder).[18] In unstructured fields, ADHD

For peer review only - http://bmjopen.bmj.com/site/about/guidelines.xhtml

diagnosis was identified and manually evaluated using previously established natural language processing techniques (PPV ~0.82).[19-22]

Educational features and other covariates

School performance indicators were collected from EYFSP and KS1 datasets in the NPD. The EYFSP comprises teacher-assessed social, linguistic, physical and cognitive development at the end of the first year of schooling.[23] KS1 comprises teacher-assessed English, maths and science at the end of the third year of formal education.[24] Gender, age, ethnicity, Free School Meal eligibility (eFSM; a proxy measure for deprivation), attendance, first language, Special Educational Need (SEN) and looked after child (LAC) status were also measured from the NPD. In total, 45 characteristics were included (Table S2 and Table S3).

Predictive model preparation and building

To minimise the potential for reverse causality, we excluded pupils who were diagnosed with ADHD prior to their KS1 assessment (n=77; 10.5% of ADHD cases). We used a complete case approach, excluding pupils with missing data from our eligible sample (n=814; 1.4%). The resulting n=56,258 formed our population cohort.

To develop and compare machine learning classifiers, the dataset was randomly divided into a training set (n=42,192; 75.0%), validation set (n=7,033; 12.5%) and test set (n=7,033; 12.5%) with a similar proportion of ADHD cases (~1%) in each set. The training set was used to train the algorithms, while the validation set was used for model refinement (e.g., hyperparameter tuning) and comparison. Final performance was evaluated on the test set. We compared several machine learning algorithms to classic statistical Logistic Regression (LR) approaches, and also a supervised approach based on neural networks, using a Multilayer Perceptron (MLP). The following classifiers were compared: LR, Random Forest (RF), Support Vector Machines (SVM), Gaussian Naive Bayes (GNB), and the MLP. For GNB, we first tested that continuous features associated with each class were distributed according to a Gaussian distribution. For the neural network, we used a four-layer architecture (input layer, two hidden layers, output layer).[25]

Selection procedure for machine learning algorithms

For peer review only - http://bmjopen.bmj.com/site/about/guidelines.xhtml

Model performances were measured with the area under the receiver operator characteristics curve (AUC). We also report precision, recall and F1-score with a fixed classification threshold of 0.5. The F1-score was calculated using the "weighted" option, which calculates metrics for each label and finds their average weighted by support. For each given value, a 95% confidence interval (CI) of the outcome measure was estimated by bootstrapping 100 times the subjects in the set. We also examined the precision-recall curve (or PR Curve) with precision (y-axis) and recall (x-axis) plotted for different probability thresholds. All the experiments with machine learning algorithms were conducted in Python with functions from the scikit learn library for classification (version 0.20.3).[26] The MLP was implemented using the Keras framework (version 2.2.4).

Tuning, resampling and ensemble methods for classification

For LR and RF, tuning of the hyperparameters was made using grid search with 10-fold cross validation (full details available by request to the author). The GNB classifier had no parameters to tune. Tuning of SVM was done empirically. To compensate for class imbalance of ADHD outcomes, we compared two different methods: random subsampling and Tomek Links.[27] For random subsampling, we successively fitted our model to multiple sub-samples. Each sample contained all ADHD cases and 1000 subjects without any ADHD diagnosis. We calculated the AUC for each fitted model and took the average value of AUC. For Tomek links, we used two approaches – removing all links from the dataset and removing the majority class samples that were part of a Tomek link. In an attempt to improve classification performance, ensemble methods were used on selected machine learning methods. We implemented Adaptative Boosting (AdaBoost) and Bagging on tuned models.[28, 29]

Selection and testing the final models

Two criteria were defined for the final models' selection: performance and transparency, i.e. the models' ability to determine feature contribution, which enabled examination of potential biases inherent to the data resources used. High performing models were considered *a priori* as having AUC ≥0.85 on the validation set.

For peer review only - http://bmjopen.bmj.com/site/about/guidelines.xhtml

Models which were both transparent and performed well were tested for diagnostic efficiency, the purpose being to assess whether the predictive performance from the population sample could generalize to clinical populations. To test the specificity of the best models for ADHD from other clinical mental health problems, we repeated our approach on a purely clinical cohort containing only the children who were present in the CRIS dataset, thus containing samples of children who presented with ADHD and non-ADHD diagnoses. Once all models were tested on the full set of features, we then refined the models to improve accuracy, reduce overfitting and increase potential for translation into busy clinical settings – making the model less vulnerable to missing information. To identify the most important features for LR models we used Beta coefficients, while RF feature importance represents the decrease in node impurity on a given branch within a decision tree, weighted by the probability of reaching that node.

Bias reduction

We investigated bias reduction for the LR and RF models trained on the population cohort using the AI Fairness 360 toolkit.[30] We used a pre-processing algorithm as we could access and modify the data directly and the data was inherently biased due to unfair clinical case ascertainment. Pre-processing algorithms learn a new representation based on protected attributes, i.e. features that partition the data set into groups that should have equal probability of getting a favourable label.[31] In our case, protected attributes were represented by ethnicity and language status, while the favourable label is an ADHD diagnosis. The Reweighing algorithm was chosen for its ability to treat two protected attributes at the same time and for the transparency of its fairness process.[32] This algorithm modified the dataset by generating different weights for the training examples in each (group, label) combination, to ensure fairness before classification.

Figure S1 displays the pre-processing fairness pipeline. We defined White and English speaking as the ‘privileged’ group, and non-White and non-English speaking as the ‘unprivileged’ group, thus obtaining two non-overlapping groups to be weighted differently. Other combinations (non-White and English speaking, White and non-English speaking) were not explicitly reweighted.

For peer review only - http://bmjopen.bmj.com/site/about/guidelines.xhtml

RESULTS

Cohort characteristics

In total, n=652 pupils in our sample were diagnosed with ADHD (1.16% of the population cohort; 15.6% of the clinical cohort) (Table 1). In the population cohort, gender was evenly balanced, and pupils were predominantly black (n=22,904, 40.71%) and spoke English as a first language (n=37,432, 66.54%). However, males were over-represented in the clinical cohort (n=2848, 68.17%), as were white ethnic groups (n=1712, 40.98%) and English first language speakers (n=3390, 81.14%). This is consistent with national surveys which report greater psychiatric morbidity among males aged 5 to 10 years, and higher rates of help-seeking and detection among white, English speaking groups.[33, 34] Compared to the population cohort, the clinical cohort also had a lower EYFSP and KS1 attainment, lower attendance, and higher rates of exclusion, eFSM, LAC and SEN.

For peer review only

For peer review only - http://bmjopen.bmj.com/site/about/guidelines.xhtml

Table 1: Characteristics of the population cohort and clinical cohort

	**Population cohort n=56258**		**Clinical cohort n=4178**	
ADHD (n, %)	652	1.16%	652	15.61%
Gender (male) (n, %)	28434	50.54%	2848	68.17%
Summer birth (n, %)	19274	34.26%	1443	34.53%
English as first language (n, %)	37432	66.54%	3390	81.14%
Ethnicity (n, %)				
Asian	4833	8.59%	129	3.09%
Black	22904	40.71%	1461	34.97%
Chinese	484	0.86%	10	0.24%
White	18218	32.38%	1712	40.98%
Mixed	7116	12.65%	667	15.97%
Other	2702	4.80%	189	4.52%
EYFSP attainment (mean, SD)				
Personal, social, & emotional development	4.98	0.99	4.32	1.20
Communication, language & literacy	4.97	0.99	4.39	1.16
Problem solving, reasoning & numeracy	4.98	0.99	4.46	1.27
Knowledge & understanding of the world	4.97	0.99	4.54	1.23
Physical development	4.98	0.99	4.45	1.31
Creative development	4.97	0.99	4.50	1.25
KS1 attainment (mean, SD)				
Maths	15.50	4.10	13.24	4.57
Reading	15.66	3.88	13.66	5.02
Writing	14.31	3.71	11.61	4.58
Science	15.23	3.55	13.29	4.43

	**EYFSP**		**KS1**		**EYFSP**		**KS1**	
eFSM (n, %)	18175	32.31%	18177	32.31%	1880	44.50 %	1882	45.05%
Looked after child status (n, %)	98	0.17%	202	0.36%	<10	<10	103	2.47%
Mainstream schooling (n, %)	-	-	55854	99.28%	-	-	4006	95.88%
Exclusion (n, %)	-	-	690	1.23 %	-	-	494	11.82%
No SEN (n, %)	45113	80.19%	45116	80.20%	2031	48.61%	2031	48.61%
Statement (n, %)	924	1.64%	922	1.64%	356	8.52%	356	8.52%
SEN school action (n, %)	5766	10.25%	5766	10.25%	675	16.16%	675	16.16%
SEN school action plus (n, %)	4454	7.92%	4453	7.92%	1116	26.71%	1116	26.71%
SEN Behavioural, social & emotional (n, %)	331	0.6%	331	0.6%	<10	<10	<10	<10

For peer review only

Model performance: identifying ADHD from population cohort

Table 2 displays AUC, Precision, Recall and F1-score, including confidence parameters for all tuned models on both validation and test sets, for classifying ADHD from the population cohort (Figure 1 for ROC analysis). Hyperparameter tuning improved RF significantly. Precision was high (above 0.985 on both the validation and test set) for LR, RF and GNB. LR, RF and the MLP all had an AUC≥0.85 on both the validation and the test set. SVM did not discriminate ADHD diagnosis as well (AUC<0.82). Overall, LR and RF were the highest performing and transparent models.

Table 2: Comparison of models: (i) Predictive performance of all trained models on the population cohort (ii) Performance of LR and RF in identifying ADHD from other child-onset psychiatric disorders within the clinical cohort. Median and 95% Confidence Interval.

Models [a]	AUC [b]		Precision		Recall		F1-score	
	Validation[c,d]	Test[c,d]	Validation[c,d]	Test[c,d]	Validation[c,d]	Test[c,d]	Validation[c,d]	Test[c,d]
**Population cohort**								
**LR**	0.862 [0.840-0.874]	0.900	0.985 [0.985-0.986]	0.987	0.835 [0.822- 0.848]	0.827	0.900 [0.892- 0.908]	0.895
**RF**	0.857 [0.842-0.869]	0.860	0.985 [0.985-0.986]	0.986	0.816 [0.802- 0.833]	0.802	0.889 [0.880-0.899]	0.880
**SVM**	0.811 [0.793-0.833]	0.830	0.980 [0.979-0.981]	0.981	0.936 [0.930-0.943]	0.908	0.957 [0.954- 0.96]	0.942
**GNB**	0.855 [0.846-0.873]	0.849	0.985 [0.985-0.986]	0.987	0.835 [0.822-0.850]	0.707	0.890 [0.882-0.909]	0.817
**MLP**	0.855 [0.834-0.864]	0.883	0.978 [0.975-0.987]	0.977	0.988 [0.982-0.990]	0.988	0.983 [0.979- 0.985]	0.983
**Clinical cohort**								
**LR**	0.718 [0.701-0.735]	0.694	0.813 [0.804-0.834]	0.797	0.659 [0.647-0.673]	0.646	0.705 [0.694-0.717]	0.693
**RF**	0.699 [0.682-0.71]	0.689	0.808 [0.784-0.823]	0.808	0.639 [0.613-0.672]	0.600	0.689 [0.665-0.715]	0.654

[a] Logistic Regression (LR), Random Forest (RF), Support Vector Machines (SVM), Gaussian Naive Bayes (GNB), Multilayer Perceptron (MLP)

[b] Area Under Curve (AUC)

[c] ADHD cases: n=82

[d] Non-ADHD cases: n=6951 in population cohort, n=441 in clinical cohort

[Figure 1: ROC curves for the tuned models]

For peer review only - http://bmjopen.bmj.com/site/about/guidelines.xhtml

Model performance: identifying ADHD from clinical cohort

Table 2 displays Precision, Recall, F1-score and final hyperparameters trained, validated and tested on the clinical cohort. Precision was ~0.80 for ADHD on both the validation and test set for both models on the clinical cohort. Both models' overall predictive performance (AUC 0.70) was much lower relative to their performance on the population cohort.

Resampling methods used to deal with class imbalance did not improve the performances significantly. Overall, Random Subsampling performed better than Tomek Links. As for ensemble approaches, a slight improvement in performance was noticed for RF only, while LR obtained better performance when using resampling methods. The precision-recall curves (PRC) for both population and clinical cohorts are displayed in Figure S2 and Figure S3. The models show significantly lower performance than ROC curve results, although they do demonstrate greater performance than a random classifier, based on PRC metrics as summarised in Table S4 comparing the PRC and ROC for LR, RF and random classification.

Bias reduction using English language and white ethnicity as protective attributes

Table 3 displays results on the population cohort (test set) when using the LR and RF models trained on transformed data (i.e., with instance weights from the fairness Reweighing algorithm). The Disparate Impact (DI) score displays the ratio of the probability of a favourable outcome for unprivileged instances and the probability of a favourable outcome for privileged instances (close to 1 in a fair dataset). The reweighing algorithm obtained a considerable improvement of the disparate impact (from 0.15-0.60 to 1), without harming the models' performance on ADHD classification: for the LR/RF models the AUC remained above 0.880/0.858 in all cases.

Table 3. Model performance on the reweighed fair dataset resulting from the bias reduction algorithm.

Models [a]	AUC	Precision	Recall	F1-score	DI before	DI after
**LR**[b]	0.880	0.986	0.810	0.885	0.149	~1.000
**RF**[b]	0.858	0.986	0.797	0.876	0.149	~1.000

[a] Logistic Regression (LR), Random Forest (RF), Disparate Impact (DI)

[b] Re-weighting model based on the 'privileged' group being both White and speaking English as a first language

For peer review only - http://bmjopen.bmj.com/site/about/guidelines.xhtml

Feature contribution for ADHD prediction in population and clinical cohorts

Table 4 displays the most important features overall, plus ethnicity, for LR and RF models in the population and clinical cohorts (all features in Table S5). KS1 writing performance was a strongly protective/discriminant factor against ADHD. Gender, KS1 attendance, SEN status, and personal, social and emotional development were also strongly discriminant features, while the importance of English language and ethnicity was reduced following reweighing.

Table 4. Feature contribution for ADHD prediction and the impact of fairness re-weighting.

	**Population cohort (n= 56257)**				**Clinical cohort (n= 4178)**	
	**LR**		**RF**		**LR**	**RF**
	**unweighted**	**weighted**	**unweighted**	**weighted**		
**KS1 Writing score**	**-0.761**	**-0.858**	**0.106**	**0.100**	**-0.407**	**0.109**
**EYFSP Personal, Social and Emotional Development**	**-0.841**	**-1.000**	**0.051**	**0.065**	**-0.314**	0.047
**KS1 Attendance (%)**	**0.958**	**1.104**	0.010	**0.008**	0.093	**0.059**
**Male gender**	0.608	**0.626**	0.029	**0.061**	**0.389**	**0.142**
**KS1 No SEN**	-0.198	-0.169	**0.146**	**0.138**	-0.094	**0.087**
**English as first language**	**0.696**	**0.183**	0.049	**0.006**	0.113	0.032
**EYFSP Attendance (%)**	**-0.983**	**-1.019**	0.010	0.005	0.093	0.050
**EYFSP Problem Solving, Reasoning and Numeracy**	**0.201**	**0.318**	0.008	**0.005**	**0.183**	0.019
**White ethnicity**	**0.224**	0.022	0.004	<0.001	0.078	0.006

Note: For the population cohort, coefficients of the model when trained on the Reweighed dataset are displayed. Feature importance metrics are different for LR and RF, and not directly comparable. LR Beta coefficients represent the log-odds of a given feature, negative values correspond with a decrease in the probability of the case being classified as ADHD and vice versa. RF feature importance is calculated as the decrease in node impurity on a given branch within a decision tree, weighted by the probability of reaching that node. The feature importance value presented represents the average feature importance over all the trees. The table displays the top 5 features for at least one model (marked in bold for the corresponding column) and top 20 features for all 4 models. The feature "White ethnicity" was added for comparing its significance after reducing bias. Ranking was made by decreasing significance. For the population cohort, coefficients of both models when trained on the Reweighed dataset using both English and White as protected attributes, are displayed.

For peer review only

DISCUSSION

This study aimed to predict pupils with a high likelihood of ADHD diagnosis using data available at the population-level. To our knowledge, it is the first study to apply machine learning approaches to large scale, routinely collected linked health and education data. These innovative methods show that ADHD in general population and clinical cohorts can be predicted with high levels of accuracy. Promisingly, this level of predictive accuracy is comparable to traditional survey-based screening methods in ADHD, and to other studies using routinely collected data to predict health outcomes.[35-38]

These findings suggest that machine learning approaches can accurately predict ADHD in large general population and clinical cohorts using existing and widely available administrative data. These models may therefore be suited to provide health intelligence to local policy makers at low cost, and inform decisions on the allocation of disorder-specific resources at an area-, school- or service-level. Tentatively, these models may also offer a means to predict ADHD at the individual-level, although greater precision would be desirable for this purpose – this is an area for future work.

We also demonstrate that sociodemographic biases inherent in the data and algorithms can be reduced with a fairness reweighing step to ensure that pupils from certain backgrounds are not systematically overlooked as being at risk of ADHD. In a geographic area with a large proportion of people from ethnic minority and non-English speaking backgrounds, minimising such biases is crucial for accurately predicting need for ADHD provision in the population.[22]

This therefore highlights the need to build fairness measures which weight for limited access to diagnosis in certain ethnic and social groups. However, it is important to note that these statistical approaches are unlikely to remove all potential biases inherent to service provision data: for example, parents also influence help-seeking for ADHD, but their characteristics are not fully captured in datasets like the NPD, and so could not be used in reweighing processes.[39] Subset scanning could be a means to detect biases in certain subgroups.[32] It should also be noted that the reweighing algorithm we used was based on the assumption that different ethnic and language groups should have equal probability of a favourable label (i.e. ADHD): under these parameters, Optimized Preprocessing could also have been applied, as it

For peer review only - http://bmjopen.bmj.com/site/about/guidelines.xhtml

ensures both group fairness and individual fairness, where similar individuals are treated similarly.[40] Further work could explore these methods.

Strengths and limitations

Our study benefited from a large and novel data linkage capturing almost all pupils from a defined geographic catchment. This reduces the risk of building models on biased and non-representative samples. These approaches are also scalable, as they use information already routinely collected by schools. However, some limitations should be highlighted. While we sought to address biases in clinical data using reweighing algorithms, biases in the education data (such as inconsistent representation of private or home-schooled pupils) have not been addressed. We also handled missing data using a complete-case analysis, which can introduce bias if the missing data are not randomly distributed, although the proportion of missing data was very low, and therefore unlikely to materially bias the findings. Nonetheless, further validation of these models is warranted.[41]

Replication in other regions or populations would also be important to assess the generalisability of these models, although at present, other educational and clinical data linkages on this scale are scarce. Finally, the similar performance across the employed machine learning algorithms may arise from imbalance within the dataset, biasing the models towards the majority class. Moreover, some of the features used in the analysis had very few observations for the ADHD cases. The potential impact of this is understudied, and warrants further investigation.[42, 43]

Conclusions

Overall, this study demonstrates that machine learning approaches using readily available education and clinical data show promise in predicting ADHD in source populations. It also highlights that biases inherent in routinely-collected data can be mitigated using fair weighting processes. With further validation and replication work, these methods have the potential to estimate burden of need for ADHD provision, thereby informing resource allocation and policy decisions.

For peer review only

For peer review only - http://bmjopen.bmj.com/site/about/guidelines.xhtml

Funding statement

JD is supported by NIHR Clinician Science Fellowship award (CS-2018-18-ST2-014) and has received support from a Medical Research Council Clinical Research Training Fellowship (MR/L017105/1) and Psychiatry Research Trust Peggy Pollak Research Fellowship in Developmental Psychiatry. SV was additionally supported by the Swedish Research Council (2015-00359), Marie Skłodowska Curie Actions, Cofund. RS, LC, JD and SV were additionally supported by a Medical Research Council Mental Health Data Pathfinder Award to King's College London. The Clinical Record Interactive Search (CRIS), RS, JD, NV and SV are part-supported by the National Institute of Health Research Biomedical Research Centre at the South London and Maudsley NHS Foundation Trust and King's College London. AW is in receipt of a PhD studentship funded by the National Institute for Health Research (NIHR) Biomedical Research Centre at South London and Maudsley NHS Foundation Trust and King's College London. The views expressed are those of the author and not necessarily those of the NHS, the NIHR or the Department of Health and Social Care. The authors are very grateful to Richard White and members of DfE National Pupil Database team, who provided invaluable support throughout the project. The funder of the study had no role in study design, data collection, data analysis, data interpretation, or writing of the report. The corresponding author had full access to all the data in the study and had final responsibility for the decision to submit for publication.

Competing interests statement

The authors report no potential conflicts of interest.

Contribution statement

The study was conceived by LTM, NV and JD. Data extraction was carried out by JD. Data analysis was undertaken by LTM and NV. Reporting of findings was led by LTM and NV with support from JD, AW, LC, SV and RS. All authors contributed to manuscript preparation and approved the final version.

For peer review only

Data sharing statement

Data can be accessed with permissions from both the Department for Education and South London and Maudsley NHS Foundation Trust.

REFERENCES

1. Rutter M. *Multiaxial classification of child and adolescent psychiatric disorders: the ICD-10 classification of mental and behavioural disorders in children and adolescents*. Cambridge University Press; 1996.
2. Polanczyk G, De Lima MS, Horta BL, Biederman J, Rohde LA. The worldwide prevalence of ADHD: a systematic review and metaregression analysis. *American journal of psychiatry*. 2007;164(6):942-948.
3. Fleming M, Fitton CA, Steiner MFC, et al. Educational and Health Outcomes of Children Treated for Attention-Deficit/Hyperactivity Disorder. *JAMA Pediatr*. Jul 3 2017;171(7):e170691. doi:10.1001/jamapediatrics.2017.0691
4. Wehmeier PM, Schacht A, Barkley RA. Social and emotional impairment in children and adolescents with ADHD and the impact on quality of life. *J Adolesc Health*. Mar 2010;46(3):209-17. doi:10.1016/j.jadohealth.2009.09.009
5. Cortese S, Adamo N, Del Giovane C, et al. Comparative efficacy and tolerability of medications for attention-deficit hyperactivity disorder in children, adolescents, and adults: a systematic review and network meta-analysis. *Lancet Psychiatry*. Sep 2018;5(9):727-738. doi:10.1016/S2215-0366(18)30269-4
6. Fridman M, Banaschewski T, Sikirica V, Quintero J, Chen KS. Access to diagnosis, treatment, and supportive services among pharmacotherapy-treated children/adolescents with ADHD in Europe: data from the Caregiver Perspective on Pediatric ADHD survey. *Neuropsychiatr Dis Treat*. 2017;13:947-958. doi:10.2147/NDT.S128752
7. Fazel M, Hoagwood K, Stephan S, Ford T. Mental health interventions in schools 1: Mental health interventions in schools in high-income countries. *Lancet Psychiatry*. Oct 2014;1(5):377-387. doi:10.1016/S2215-0366(14)70312-8
8. Weist MD, Rubin M, Moore E, Adelsheim S, Wrobel G. Mental health screening in schools. *J Sch Health*. Feb 2007;77(2):53-8. doi:10.1111/j.1746-1561.2007.00167.x
9. Heller MD, Roots K, Srivastava S, Schumann J, Srivastava J, Hale TS. A Machine Learning-Based Analysis of Game Data for Attention Deficit Hyperactivity Disorder Assessment. *Games Health J*. Oct 2013;2(5):291-8. doi:10.1089/g4h.2013.0058
10. Sen B, Borle NC, Greiner R, Brown MRG. A general prediction model for the detection of ADHD and Autism using structural and functional MRI. *PLoS One*. 2018;13(4):e0194856. doi:10.1371/journal.pone.0194856
11. Sun H, Chen Y, Huang Q, et al. Psychoradiologic Utility of MR Imaging for Diagnosis of Attention Deficit Hyperactivity Disorder: A Radiomics Analysis. *Radiology*. May 2018;287(2):620-630. doi:10.1148/radiol.2017170226
12. Tenev A, Markovska-Simoska S, Kocarev L, Pop-Jordanov J, Muller A, Candrian G. Machine learning approach for classification of ADHD adults. *Int J Psychophysiol*. Jul 2014;93(1):162-6. doi:10.1016/j.ijpsycho.2013.01.008
13. Rajkomar A, Hardt M, Howell MD, Corrado G, Chin MH. Ensuring Fairness in Machine Learning to Advance Health Equity. *Ann Intern Med*. Dec 18 2018;169(12):866-872. doi:10.7326/M18-1990

For peer review only - http://bmjopen.bmj.com/site/about/guidelines.xhtml

14. Coker TR, Elliott MN, Toomey SL, et al. Racial and Ethnic Disparities in ADHD Diagnosis and Treatment. *Pediatrics*. Sep 2016;138(3)doi:10.1542/peds.2016-0407
15. Jay MA, McGrath-Lone L, Gilbert R. Data Resource: the National Pupil Database (NPD). *Int J Popul Data Sci*. Mar 20 2019;4(1):1101. doi:10.23889/ijpds.v4i1.1101
16. Downs J, Gilbert R, Hayes RD, Hotopf M, Ford T. Linking health and education data to plan and evaluate services for children. *Arch Dis Child*. Jul 2017;102(7):599-602. doi:10.1136/archdischild-2016-311656
17. Downs JM, Ford T, Stewart R, et al. An approach to linking education, social care and electronic health records for children and young people in South London: a linkage study of child and adolescent mental health service data. *BMJ Open*. Jan 29 2019;9(1):e024355. doi:10.1136/bmjopen-2018-024355
18. World Health Organization. *International Statistical Classification of Diseases and Related Health Problems*. 10th Revision ed. 2000.
19. Downs J, Hotopf M, Ford T, et al. Clinical predictors of antipsychotic use in children and adolescents with autism spectrum disorders: a historical open cohort study using electronic health records. *Eur Child Adolesc Psychiatry*. Jun 2016;25(6):649-58. doi:10.1007/s00787-015-0780-7
20. Jackson RG, Patel R, Jayatilleke N, et al. Natural language processing to extract symptoms of severe mental illness from clinical text: the Clinical Record Interactive Search Comprehensive Data Extraction (CRIS-CODE) project. *BMJ Open*. Jan 17 2017;7(1):e012012. doi:10.1136/bmjopen-2016-012012
21. O'Connor C, Downs J, McNicholas F, Cross L, Shetty H. Documenting diagnosis in child and adolescent mental healthcare: A content analysis of diagnostic statements in a psychiatric case register. *Children and Youth Services Review*. 2020;113:104948.
22. Perera G, Broadbent M, Callard F, et al. Cohort profile of the South London and Maudsley NHS Foundation Trust Biomedical Research Centre (SLaM BRC) Case Register: current status and recent enhancement of an Electronic Mental Health Record-derived data resource. *BMJ Open*. Mar 1 2016;6(3):e008721. doi:10.1136/bmjopen-2015-008721
23. Early years foundation stage profile handbook. . GOV.UK. Accessed 15 Sep 2019, https://www.gov.uk/government/publications/early-years-foundation-stage-profile-handbook
24. Key stage 1 teacher assessment guidance. GOV.UK. Accessed 15 Sep 2019, https://www.gov.uk/government/publications/key-stage-1-teacher-assessment-guidance
25. Bebis G, Georgiopoulos M. Feed-forward neural networks. *IEEE Potentials*. 1994;13(4):27-31.
26. scikit-learn: machine learning in Python — scikit-learn 0.21.3 documentation. Accessed 15 Sep 2019, https://scikit-learn.org/stable/
27. Two Modifications of CNN. *IEEE Transactions on Systems, Man, and Cybernetics*. 1976;SMC-6(11):769-772. doi:10.1109/TSMC.1976.4309452
28. Breiman L. Bagging predictors. *Machine learning*. 1996;24(2):123-140.
29. Schapire RE, Freund Y. Boosting: Foundations and algorithms. *Kybernetes*. 2013;
30. Bellamy RK, Dey K, Hind M, et al. AI Fairness 360: An extensible toolkit for detecting and mitigating algorithmic bias. *IBM Journal of Research and Development*. 2019;63(4/5):4: 1-4: 15.
31. d'Alessandro B, O'Neil C, LaGatta T. Conscientious Classification: A Data Scientist's Guide to Discrimination-Aware Classification. *Big Data*. Jun 2017;5(2):120-134. doi:10.1089/big.2016.0048
32. Zhang Z, Neill DB. Identifying significant predictive bias in classifiers. *arXiv preprint arXiv:161108292*. 2016;

For peer review only - http://bmjopen.bmj.com/site/about/guidelines.xhtml

33. Ford T, Goodman R, Meltzer H. The British Child and Adolescent Mental Health Survey 1999: the prevalence of DSM-IV disorders. *J Am Acad Child Adolesc Psychiatry*. Oct 2003;42(10):1203-11. doi:10.1097/00004583-200310000-00011
34. Ford T, Hamilton H, Meltzer H, Goodman R. Predictors of Service Use for Mental Health Problems Among British Schoolchildren. *Child Adolesc Ment Health*. Feb 2008;13(1):32-40. doi:10.1111/j.1475-3588.2007.00449.x
35. Algorta GP, Dodd AL, Stringaris A, Youngstrom EA. Diagnostic efficiency of the SDQ for parents to identify ADHD in the UK: a ROC analysis. *Eur Child Adolesc Psychiatry*. Sep 2016;25(9):949-57. doi:10.1007/s00787-015-0815-0
36. Basu S, Narayanaswamy R. A Prediction Model for Uncontrolled Type 2 Diabetes Mellitus Incorporating Area-level Social Determinants of Health. *Med Care*. Aug 2019;57(8):592-600. doi:10.1097/MLR.0000000000001147
37. Hammond R, Athanasiadou R, Curado S, et al. Predicting childhood obesity using electronic health records and publicly available data. *PLoS One*. 2019;14(4):e0215571. doi:10.1371/journal.pone.0215571
38. Xiao C, Choi E, Sun J. Opportunities and challenges in developing deep learning models using electronic health records data: a systematic review. *J Am Med Inform Assoc*. Oct 1 2018;25(10):1419-1428. doi:10.1093/jamia/ocy068
39. Sayal K, Prasad V, Daley D, Ford T, Coghill D. ADHD in children and young people: prevalence, care pathways, and service provision. *The Lancet Psychiatry*. 2018;5(2):175-186.
40. Neill DB. Expectation-based scan statistics for monitoring spatial time series data. *International Journal of Forecasting*. 2009;25(3):498-517.
41. Riley RD, Ensor J, Snell KI, et al. External validation of clinical prediction models using big datasets from e-health records or IPD meta-analysis: opportunities and challenges. *BMJ*. Jun 22 2016;353:i3140. doi:10.1136/bmj.i3140
42. Johnson JM, Khoshgoftaar TM. Survey on deep learning with class imbalance. *Journal of Big Data*. 2019/03/19 2019;6(1):27. doi:10.1186/s40537-019-0192-5
43. van der Ploeg T, Austin PC, Steyerberg EW. Modern modelling techniques are data hungry: a simulation study for predicting dichotomous endpoints. *BMC Med Res Methodol*. Dec 22 2014;14:137. doi:10.1186/1471-2288-14-137

For peer review only

For peer review only - http://bmjopen.bmj.com/site/about/guidelines.xhtml

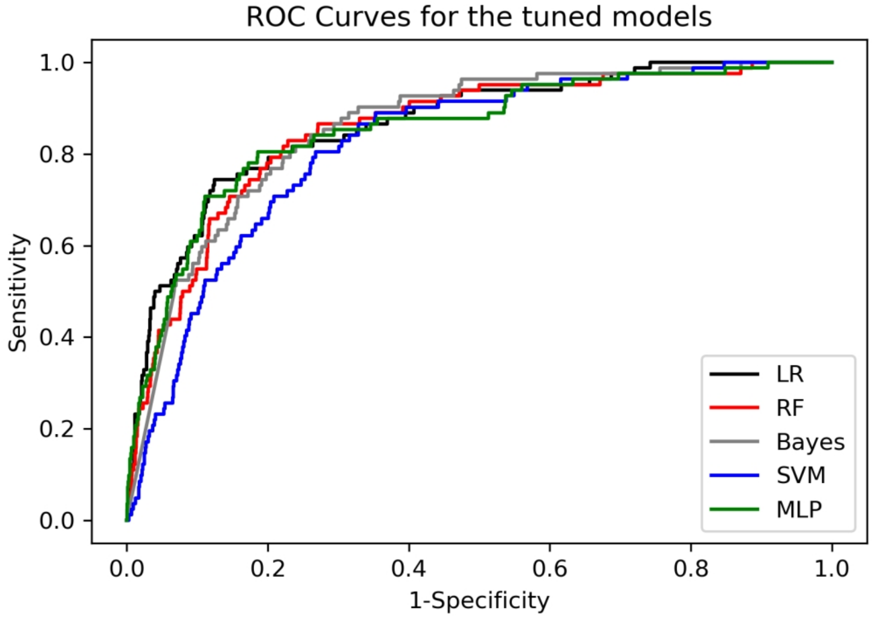

Figure 1: ROC curves for the tuned models

387x258mm (118 x 118 DPI)

Table S2: Educational Features

	Feature name	Feature Description
Demographic features	Gender (Male)	Dummy variable indicating male gender.
	Age	Age in years and months at the start of KS1.
	Summer birth	Variable indicating whether the child was born in summer months of school term (1st April- 31st August). These are the youngest children in an academic cohort.
	Ethnicity	Dummy variables indicating ethnic background. We included 6 separate ethnicity dummy variables within the model: Asian, Black, Chinese, White, Mixed, Other.
	Deprivation Indicator (IDACI)	The Income Deprivation Affecting Children Index (IDACI) scores are an indicator of deprivation. The IDACI measures the proportion of all children aged 0 to 15 living in income deprived families. It is a subset of the Income Deprivation Domain which measures the proportion of the population in an area experiencing deprivation relating to low income.
	Academic Year	This feature describes the academic year a child started KS1 (for example 2011).
Additional need indicators	eFSM	Eligibility for Free School Meals is a statutory benefit available to school aged children in England from less affluent backgrounds. eFSM is a commonly used proxy variable for lower socio-economic status.
	Mobility (KS1)	Variable indicating whether a child has moved schools within KS1.
	Mobility (ever)	Variable indicating whether a child has moved schools ever (i.e. during KS1 or the EYFSP).
	Child Looked After Status	Child looked after status indicates that a child has been in the care of their local authority for more than 24 hours.
	English first language	English as a first language refers to whether the child's native language is English or other.
Special Educational Need indicators	No SEN	Dummy variable indicating that the child had no Special Educational Needs.
	SEN School Action	Dummy variable indicating that a child has a school action plan to support their SEN. This might include adapted teaching methods and materials, or in some cases additional adult support. This plan is created by the school.
	SEN School Action+	Dummy variable indicating that a child has had a school action plan to support their SEN, however this has not helped the child make adequate progress. At this point school action plus is required. This involves schools consulting and collaborating with appropriate external support services (for example, a speech and language therapist).
	SEN Statement of needs	Dummy variable indicating that child has a SEN statement of needs. A SEN Statement is a plan set by the Education Authority detailing a child's needs and required support. This is typically made if a child needs support beyond the scope of the school (school action/ school action plus).
	SEN: Autism spectrum disorders	Autism spectrum disorder is a specific SEN. It is a diagnosable disorder that affects social interaction, interests and behaviour.
	SEN: specific learning difficulty	Children with specific learning difficulties, typically struggle with one specific aspect of learning but are able in other areas. For

For peer review only - http://bmjopen.bmj.com/site/about/guidelines.xhtml

		example, they may find reading particularly challenging (but cope well with numbers and letters).
	SEN: moderate learning difficulty	Learning difficulties are formally diagnosed by a clinician. The SEN: moderate learning difficulty code indicates a child's ability to understand and communicate information is moderately impaired.
	SEN: severe learning difficulty	Learning difficulties are formally diagnosed by a clinician. The SEN: severe learning difficulty code indicates a child's ability to understand and communicate information is severely impaired.
	SEN: behavioural, emotional and social difficulties	SEN: Behavioural, social and emotional difficulties is a broad term referring to children who's behaviour poses a barrier to learning. This can be vary in terms of presentation, children may be withdrawn or isolated, disruptive, lack concentration, immature social skills, or other challenging behaviours.
	SEN: speech, language & communication needs	This variable highlights those who find it difficult to listen, understand or communicate with others that adversely affects educational performance.
	SEN: hearing impairment	This variable indicates a child who has either a permanent or fluctuating hearing impairment that adversely affects educational performance.
	SEN: vision impairment	This variable indicates a child who has either a permanent or fluctuating visual impairment that adversely affects educational performance.
	SEN: multi-sensory impairment	This variable indicates a child who has either a permanent or fluctuating multi-sensory impairment (e.g. hearing and vison) that adversely affects educational performance.
	SEN: physical	This variable indicates a child who has either a permanent or fluctuating physical impairment that adversely affects educational performance.
	SEN: other	This variable indicates a child who has a special educational need which is adversely affecting education performance, but not captured in the categories above.
	Mainstream education indicator	Dummy variable indicating that a child is in mainstream education (as opposed to alternative provision).
School attendance and exclusion features	Attendance (%)	The percentage of time within the school year that a child was registered as attending school.
	Exclusion (temporary)	A fixed period exclusion where a child is temporarily moved from school (a maximum of 45 days within a school year).
	Exclusion (permanent)	Permanent exclusion indicating a child has been expelled from school.
School attainment features	EYFSP knowledge of the world	EYFSP sub scale: assesses a child's ability to make sense of their physical world and community on a scale of 1-9 (with 9 being most proficient). This is not a standardised assessment, however it is conducted by a trained professional (Early Years Practitioner) and moderated by the local authority.
	EYFSP problem solving, reasoning and numeracy	EYFSP sub scale: assesses a child's ability to problem solve, reason and perform basic counting and calculations on a scale of 1-9 (with 9 being most proficient). This is not a standardised assessment, however it is conducted by a trained professional (Early Years Practitioner) and moderated by the local authority.

For peer review only - http://bmjopen.bmj.com/site/about/guidelines.xhtml

	EYFSP personal, social and emotional development	EYFSP sub scale: assesses a child's personal, social and emotional development on a scale of 1-9 (with 9 being most proficient). This is not a standardised assessment, however it is conducted by a trained professional (Early Years Practitioner) and moderated by the local authority.
	EYFSP communication, language and literacy	EYFSP sub scale: assesses a child's communication, language and literacy performance on a scale of 1-9 (with 9 being most proficient). This is not a standardised assessment, however it is conducted by a trained professional (Early Years Practitioner) and moderated by the local authority.
	EYFSP physical development	EYFSP sub scale: assesses a child's physical development on a scale of 1-9 (with 9 being most proficient). This is not a standardised assessment, however it is conducted by a trained professional (Early Years Practitioner) and moderated by the local authority.
	EYFSP creative development	EYFSP sub scale: assesses a child's creative development on a scale of 1-9 (with 9 being most proficient). This is not a standardised assessment, however it is conducted by a trained professional (Early Years Practitioner) and moderated by the local authority.
	KS1 Maths	National Standardised Test: Mathematics assessment score.
	KS1 Writing	National Standardised Test: Writing assessment score.
	KS1 Reading	National Standardised Test: Reading assessment score.
	KS1 Science	National Standardised Test: Science assessment score.

Note: Educational features are drawn from the National Pupil Database (NPD). The NPD is a longitudinal database which holds individual-level records for all children and young people enrolled in state school, or taking national exams, and are currently residing in England. The database is large, with over 15 million records added since it's introduction (2002). The NPD holds information on a child's attainment, attendance, school context, and level of need (in addition to demographic variables). Data is provided on a quarterly basis by all state funded primary and secondary schools across England, via a dedicated submission system to the government's Department for Education.

For peer review only

Table S3: Educational feature time points

		EYFSP	KS1
Demographic features	Gender (Male)	✓	✓
	Age	✓	✓
	Summer Birth	✓	✓
	Ethnicity	✓	✓
	Deprivation Indicator (IDACI)	✓	✓
	Academic Year		✓
Additional need indicators	eFSM	✓	✓
	Mobility (KS1)		✓
	Mobility (ever)		✓
	Child Looked After Status	✓	✓
	English first language	✓	✓
Special Educational Need indicators	No SEN	✓	✓
	SEN Statement	✓	✓
	SEN School Action	✓	✓
	SEN School Action+	✓	✓
	SEN: Autism spectrum disorders	✓	✓
	SEN: specific learning difficulty	✓	✓
	SEN: moderate learning difficulty	✓	✓
	SEN: severe learning difficulties	✓	✓
	SEN: behavioural, emotional and social difficulty	✓	✓
	SEN: speech, language & communication needs	✓	✓
	SEN: hearing impairment	✓	✓
	SEN: vision impairment	✓	✓
	SEN: multi-sensory impairment	✓	✓
	SEN: physical	✓	✓
	SEN: other	✓	✓
	Mainstream education indicator		✓
School attendance and exclusion features	Attendance (%)	✓	✓
	Excluded (temporary)		✓
	Excluded (permanent)		✓
School attainment features	EYFSP knowledge of the world	✓	
	EYFSP problem solving, reasoning and numeracy	✓	
	EYFSP personal, social and emotional development	✓	
	EYFSP communication, language and literacy	✓	
	EYFSP physical development	✓	
	EYFSP creative development	✓	
	KS1 Maths		✓
	KS1 Writing		✓
	KS1 Reading		✓
	KS1 Science		✓

For peer review only - http://bmjopen.bmj.com/site/about/guidelines.xhtml

Figure S1: Pre-processing fairness pipeline

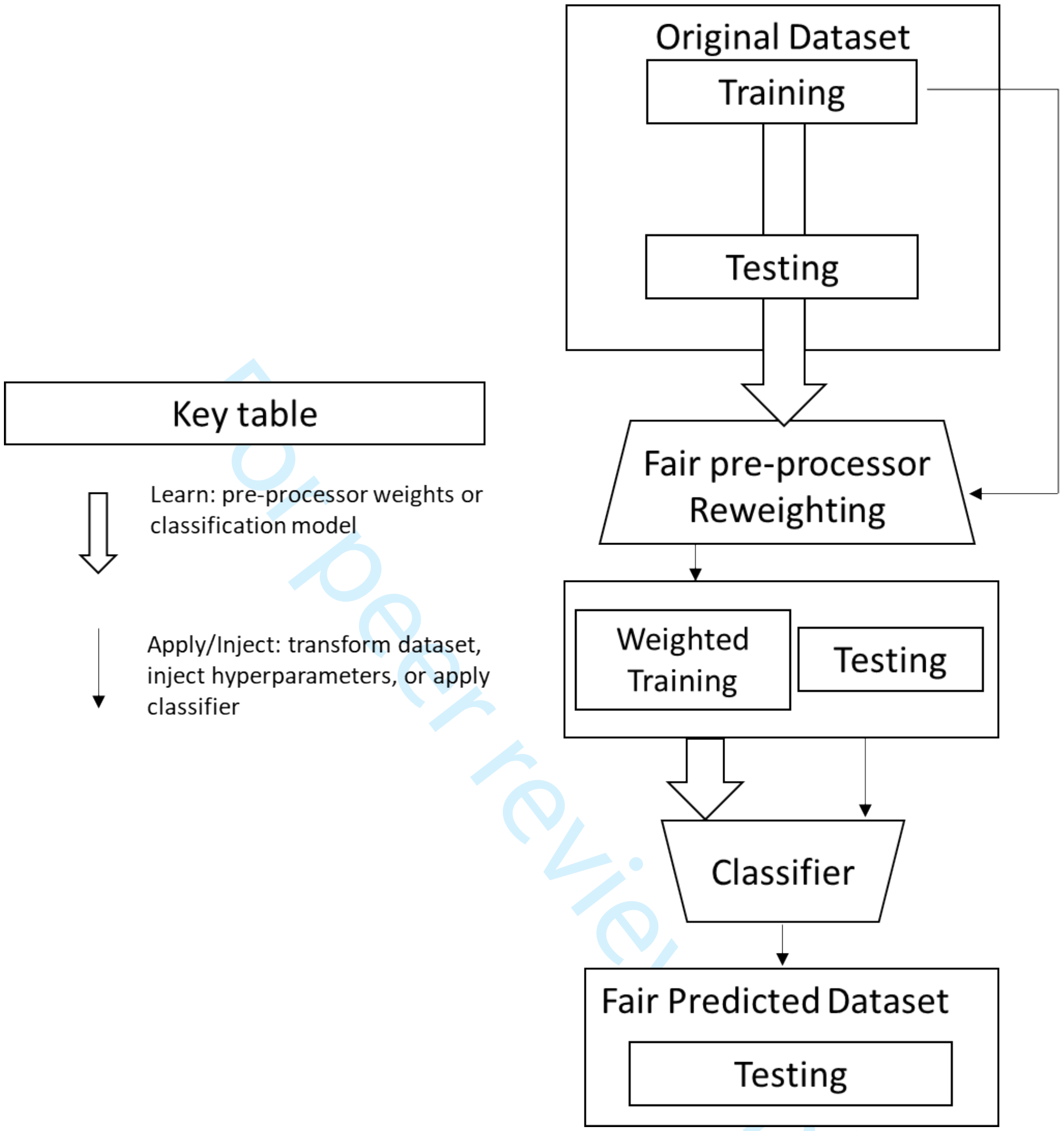

For peer review only - http://bmjopen.bmj.com/site/about/guidelines.xhtml

Figure S2: Precision-Recall curves comparing Logistic Regression(LR), Random Forest (RF) and a Random classifier for ADHD diagnosis within a population cohort

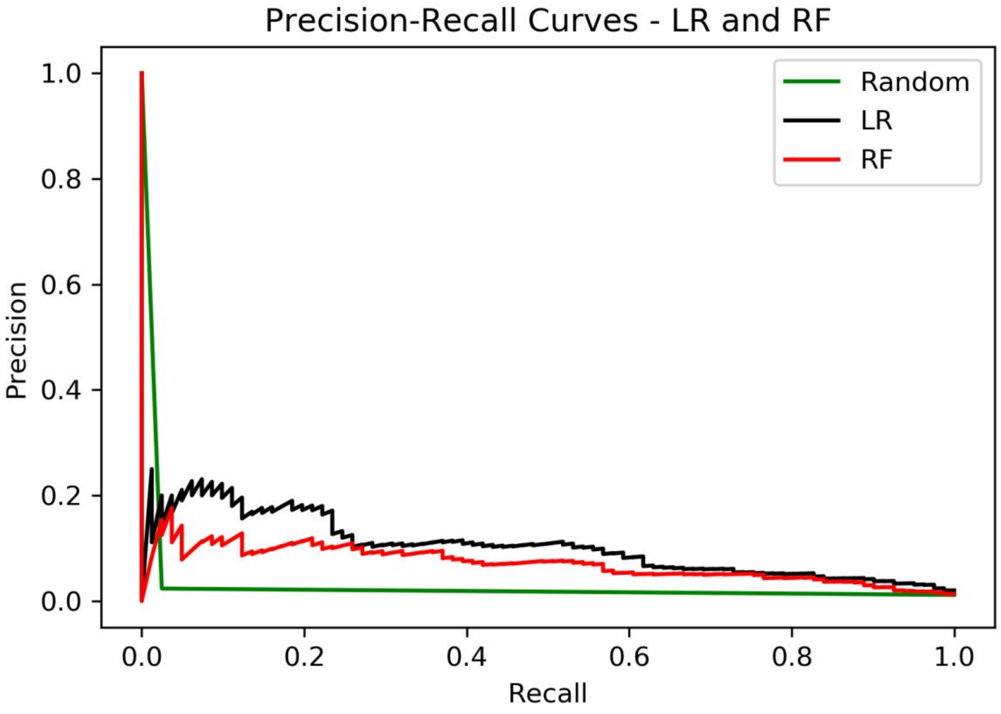

Figure S3: Precision-Recall curves comparing Logistic Regression(LR), Random Forest(RF) and a Random classifier for ADHD diagnosis within a clinical cohort

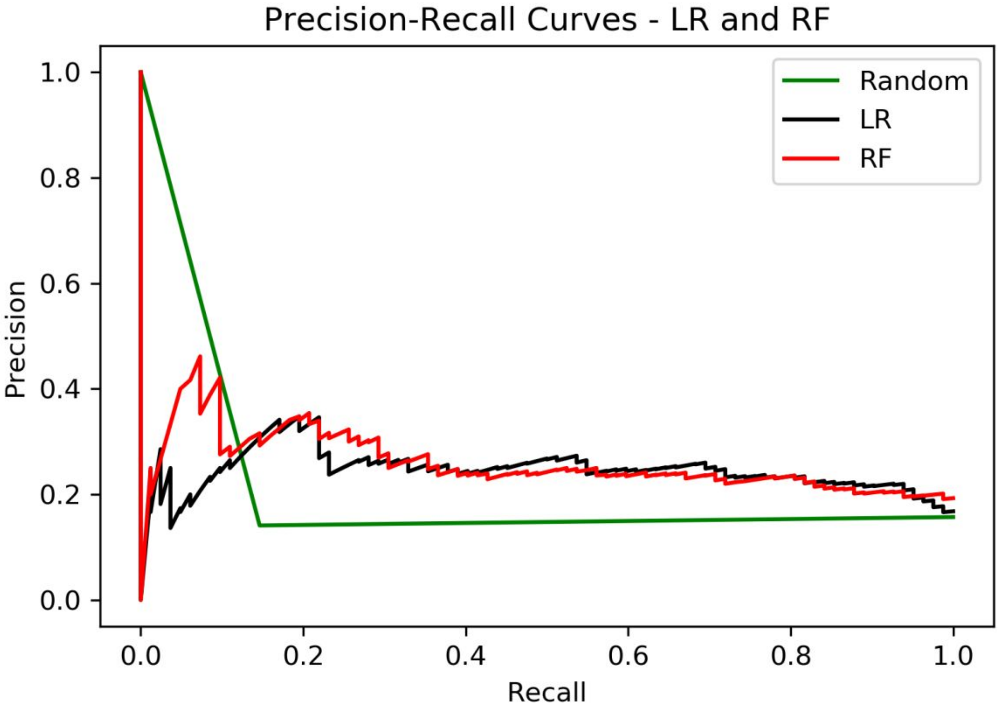

For peer review only - http://bmjopen.bmj.com/site/about/guidelines.xhtml

Table S4: AUC comparison between ROC and PRC analyses for population and clinical classification of ADHD.

	Population cohort		Clinical cohort	
**Classification Model**	ROC[c] AUC[d]	PRC[e] AUC[d]	ROC[c] AUC[d]	PRC[e] AUC[d]
**LR[a]**	0.900	0.101	0.694	0.244
**RF[b]**	0.86	0.070	0.688	0.257
**Random**	0.50	0.029	0.50	0.21

[a] Logistic Regression (LR), [b]Random Forest (RF), [c] Receiver Operating Characteristic curve (ROC)
[d] Area Under Curve (AUC) [e] Precision-recall curve

For peer review only

Table S4: Pupil characteristics and feature contribution (unweighted) for ADHD prediction in the general population sample.

Characteristics	Logistic Regression (***B*** co-efficient)	Random Forest (feature importance)
Male Gender	0.608463661	0.141773129
Summer Born (May-August)	0.109707469	0.000807359
White Ethnic group	0.201065669	0.006387057
Black Ethnic group	-0.098073916	0.000345206
Asian Ethnic Group	-0.15535868	0.011452877
Chinese Ethnic group	-0.469369952	0
Mixed Ethnic group	0.165278809	0.001506941
Other Ethnic Group	-0.065271609	0.002849315
English as first language	0.696034466	0.031602634
EYFSP: FSM eligible	0.110110336	0.001856049
EYFSP: Looked After Child	-0.058157067	0
EYFSP: IDACI score	-0.245109352	0.012435495
EYFSP: Communication, Language and Literacy	0.180721245	0.01633496
EYFSP: Knowledge of the World z score	0.436711733	0.011483452
EYFSP: Person, social, emotion z score	-0.84127724	0.047292232
EYFSP: Physical Development	-0.027708196	0.007938476
EYFSP: Creative Development	0.123449383	0.01211572
EYFSP: Problem solving, reasoning and numeracy	0.224475588	0.019006476
school mobility (ever)	-0.066303394	0.001939589
EYFSP: attendance (%)	-0.982714854	0.050421392
EYSFSP local school	-0.077583539	0.0017516
EYSFSP school type	-0.074490398	0.003640833
EYFSP: No SEN	-0.105148801	0.093914996
EYFSP: SEN School Action	0.048150906	0.008735734
EYFSP: SEN School Action Plus	0.122221511	0.030373947
EYFSP: SEN Statement	-0.044818697	0
EYFSP: SEN speech, language & communication needs	-0.113501845	0.001918025
EYFSP: SEN Autism	-0.027233555	0
EYFSP: SEN behavioural, emotional and social difficulty	-0.087858945	0.005794567
EYFSP: SEN Hearing	0.002234986	0.002198098
EYFSP: SEN Moderate learning difficulty	-0.002195983	0.000564267
EYFSP: SEN Other	0.046557778	0.00206679
EYFSP: SEN Physical	-0.043438673	0.003612997
EYFSP: SEN Specific learning difficulty	0.008096732	0
EYFSP: SEN Severe learning difficulty	-0.087701227	0.000828542
EYFSP: SEN Vision	-0.014857814	0
EYFSP: SEN Multi-sensory impairment	-0.137831643	0
KS1: Age at start	0.100899579	0.018690101
KS1 : Academic Year (e.g. 2008/9)	-0.046794005	0.022486708
KS1: mainstream school	-0.064770905	0.002768431

For peer review only

KS1: FSM eligible	0.113473204	0.001361182
KS1: IDACI score	0.17501001	0.006385568
KS1: Looked After Child	0.054056081	0.001767296
KS1: Maths score	-0.164521477	0.032731843
KS1: Writing point score	-0.761201837	0.10916803
KS1: Read Score	0.001961117	0.035857909
KS1: Science score	-0.028166159	0.010063037
KS1 school mobility	0.054731598	0.001614064
KS1: attendance (%)	0.957564403	0.059408817
KS1: Excluded	0.125124512	0.004965946
KS1: Exclusion	0.024925737	0.005752298
KS1: Not SEN	-0.198059867	0.08701934
KS1: SEN School Action	0.063043447	0.003333059
KS1: SEN status: school action plus	0.175904692	0.020499615
KS1: SEN: speech, language & communication needs	-0.030230103	0.000800672
KS1: SEN Autism	0.147169063	0.002138406
KS1: SEN behavioural, emotional and social difficulty	-0.087858945	0.00538704
KS1: SEN Hearing	0.002234986	0.001026292
KS1: SEN Moderate learning difficulty	-0.002195983	0.000752458
KS1: SEN Multi-sensory impairment	-0.137831643	0
KS1: SEN Other	0.046557778	0.007853115
KS1: SEN Physical	-0.043438673	0.004452396
KS1: SEN Specific learning difficulty	0.008096732	0.000377725
KS1: SEN Severe Learning Difficulty	0.043312749	0
KS1: SEN Statement	0.09702835	0
KS1: SEN Vision	-0.014857814	0

For peer review only

Table S1: The RECORD statement – checklist of items, extended from the STROBE statement, that should be reported in observational studies using routinely collected health data. Items specific to cross-sectional/case-control studies have been removed.

	Item No.	STROBE items	Location in manuscript where items are reported	RECORD items	Location in manuscript where items are reported
**Title and abstract**					
	1	(a) Indicate the study's design with a commonly used term in the title or the abstract (b) Provide in the abstract an informative and balanced summary of what was done and what was found	3	RECORD 1.1: The type of data used should be specified in the title or abstract. When possible, the name of the databases used should be included.<br><br>RECORD 1.2: If applicable, the geographic region and timeframe within which the study took place should be reported in the title or abstract.<br><br>RECORD 1.3: If linkage between databases was conducted for the study, this should be clearly stated in the title or abstract.	3
**Introduction**					
Background rationale	2	Explain the scientific background and rationale for the investigation being reported	4-5	N/A	
Objectives	3	State specific objectives, including any prespecified hypotheses	5	N/A	
**Methods**					
Study Design	4	Present key elements of study design early in the paper	5-6	N/A	
Setting	5	Describe the setting, locations, and relevant dates, including periods of recruitment, exposure, follow-up, and data collection	3, 5-6	N/A	
Participants	6	*(a) Cohort study* - Give the eligibility criteria, and the sources and methods of selection of participants. Describe methods of follow-up	6	RECORD 6.1: The methods of study population selection (such as codes or algorithms used to identify subjects) should be listed in detail. If this is not possible, an explanation should be provided.	6

For peer review only

		(b) Cohort study - For matched studies, give matching criteria and number of exposed and unexposed		RECORD 6.2: Any validation studies of the codes or algorithms used to select the population should be referenced. If validation was conducted for this study and not published elsewhere, detailed methods and results should be provided.<br><br>RECORD 6.3: If the study involved linkage of databases, consider use of a flow diagram or other graphical display to demonstrate the data linkage process, including the number of individuals with linked data at each stage.	
Variables	7	Clearly define all outcomes, exposures, predictors, potential confounders, and effect modifiers. Give diagnostic criteria, if applicable.	6-7	RECORD 7.1: A complete list of codes and algorithms used to classify exposures, outcomes, confounders, and effect modifiers should be provided. If these cannot be reported, an explanation should be provided.	6-7
Data sources/ measurement	8	For each variable of interest, give sources of data and details of methods of assessment (measurement). Describe comparability of assessment methods if there is more than one group	6-7	N/A	
Bias	9	Describe any efforts to address potential sources of bias	9	N/A	
Study size	10	Explain how the study size was arrived at	7	N/A	
Quantitative variables	11	Explain how quantitative variables were handled in the analyses. If applicable, describe which groupings were chosen, and why	6-7	N/A	
Statistical methods	12	(a) Describe all statistical methods, including those used to control for confounding<br>(b) Describe any methods used to examine subgroups and interactions<br>(c) Explain how missing data were addressed	7-9	N/A	

For peer review only

		(d) *Cohort study* - If applicable, explain how loss to follow-up was addressed<br>(e) Describe any sensitivity analyses			
Data access and cleaning methods		N/A		RECORD 12.1: Authors should describe the extent to which the investigators had access to the database population used to create the study population.<br><br>RECORD 12.2: Authors should provide information on the data cleaning methods used in the study.	6
Linkage		N/A		RECORD 12.3: State whether the study included person-level, institutional-level, or other data linkage across two or more databases. The methods of linkage and methods of linkage quality evaluation should be provided.	5-6
**Results**					
Participants	13	(a) Report the numbers of individuals at each stage of the study (*e.g.*, numbers potentially eligible, examined for eligibility, confirmed eligible, included in the study, completing follow-up, and analysed)<br>(b) Give reasons for non-participation at each stage.<br>(c) Consider use of a flow diagram	7	RECORD 13.1: Describe in detail the selection of the persons included in the study (*i.e.,* study population selection) including filtering based on data quality, data availability and linkage. The selection of included persons can be described in the text and/or by means of the study flow diagram.	6-7
Descriptive data	14	(a) Give characteristics of study participants (*e.g.*, demographic, clinical, social) and information on exposures and potential confounders<br>(b) Indicate the number of participants with missing data for each variable of interest<br>(c) *Cohort study* - summarise follow-up time (*e.g.*, average and total amount)	10-11	N/A	
Outcome data	15	*Cohort study* - Report numbers of outcome events or summary measures over time	10-11	N/A	
Main results	16	(a) Give unadjusted estimates and, if applicable, confounder-adjusted	N/A	N/A	

For peer review only

		estimates and their precision (e.g., 95% confidence interval). Make clear which confounders were adjusted for and why they were included<br>(b) Report category boundaries when continuous variables were categorized<br>(c) If relevant, consider translating estimates of relative risk into absolute risk for a meaningful time period			
Other analyses	17	Report other analyses done—e.g., analyses of subgroups and interactions, and sensitivity analyses	12-14	N/A	
**Discussion**					
Key results	18	Summarise key results with reference to study objectives	15	N/A	
Limitations	19	Discuss limitations of the study, taking into account sources of potential bias or imprecision. Discuss both direction and magnitude of any potential bias	16	RECORD 19.1: Discuss the implications of using data that were not created or collected to answer the specific research question(s). Include discussion of misclassification bias, unmeasured confounding, missing data, and changing eligibility over time, as they pertain to the study being reported.	16
Interpretation	20	Give a cautious overall interpretation of results considering objectives, limitations, multiplicity of analyses, results from similar studies, and other relevant evidence	16	N/A	
Generalisability	21	Discuss the generalisability (external validity) of the study results	16	N/A	
**Other Information**					
Funding	22	Give the source of funding and the role of the funders for the present study and, if applicable, for the original study on which the present article is based	17	N/A	
Accessibility of protocol, raw data, and programming code		N/A		RECORD 22.1: Authors should provide information on how to access any supplemental information such as the study protocol, raw data, or programming code.	18

For peer review only

*Reference: Benchimol, E. I., Smeeth, L., Guttmann, A., Harron, K., Moher, D., Petersen, I., ... & RECORD Working Committee. (2015). The REporting of studies Conducted using Observational Routinely-collected health Data (RECORD) statement. PLoS Med, 12(10), e1001885.

*Checklist is protected under Creative Commons Attribution (CC BY) license.

For peer review only

BMJ Open

Assessing machine learning for fair prediction of ADHD in school pupils using a retrospective cohort study of linked education and healthcare data

Journal:	*BMJ Open*
Manuscript ID	bmjopen-2021-058058.R1
Article Type:	Original research
Date Submitted by the Author:	27-May-2022
Complete List of Authors:	Ter-Minassian, Lucile; King's College London, Department of Psychological Medicine<br>Viani, Natalia; King's College London, Department of Psychological Medicine<br>Wickersham, Alice; King's College London, Institute of Psychiatry, Psychology and Neuroscience<br>Cross, Lauren; King's College London, Department of Psychological Medicine<br>Stewart, Robert; King's College London, Institute of Psychiatry<br>Velupillai, Sumithra; Kings College London, Institute of Psychiatry, Psychology and Neuroscience<br>Downs, Johnny; South London and Maudsley NHS Foundation Trust, Department of Child and Adolescent Psychiatry
**Primary Subject Heading**:	Mental health
Secondary Subject Heading:	Public health
Keywords:	Child & adolescent psychiatry < PSYCHIATRY, EPIDEMIOLOGY, MENTAL HEALTH

SCHOLARONE™
Manuscripts

I, the Submitting Author has the right to grant and does grant on behalf of all authors of the Work (as defined in the below author licence), an exclusive licence and/or a non-exclusive licence for contributions from authors who are: i) UK Crown employees; ii) where BMJ has agreed a CC-BY licence shall apply, and/or iii) in accordance with the terms applicable for US Federal Government officers or employees acting as part of their official duties; on a worldwide, perpetual, irrevocable, royalty-free basis to BMJ Publishing Group Ltd ("BMJ") its licensees and where the relevant Journal is co-owned by BMJ to the co-owners of the Journal, to publish the Work in this journal and any other BMJ products and to exploit all rights, as set out in our licence.

The Submitting Author accepts and understands that any supply made under these terms is made by BMJ to the Submitting Author unless you are acting as an employee on behalf of your employer or a postgraduate student of an affiliated institution which is paying any applicable article publishing charge ("APC") for Open Access articles. Where the Submitting Author wishes to make the Work available on an Open Access basis (and intends to pay the relevant APC), the terms of reuse of such Open Access shall be governed by a Creative Commons licence – details of these licences and which Creative Commons licence will apply to this Work are set out in our licence referred to above.

Other than as permitted in any relevant BMJ Author's Self Archiving Policies, I confirm this Work has not been accepted for publication elsewhere, is not being considered for publication elsewhere and does not duplicate material already published. I confirm all authors consent to publication of this Work and authorise the granting of this licence.

For peer review only

Title

Assessing machine learning for fair prediction of ADHD in school pupils using a retrospective cohort study of linked education and healthcare data

Author names and affiliations

Ms Lucile Ter-Minassian, MSc

Department of Psychological Medicine, Institute of Psychiatry, Psychology & Neuroscience, King's College London, London, UK.

Dr Natalia Viani*, PhD

Department of Psychological Medicine, Institute of Psychiatry, Psychology & Neuroscience, King's College London, London, UK.

Ms Alice Wickersham*, MSc

Department of Psychological Medicine, Institute of Psychiatry, Psychology & Neuroscience, King's College London, London, UK.

Ms Lauren Cross*, MSc

Department of Psychological Medicine, Institute of Psychiatry, Psychology & Neuroscience, King's College London, London, UK.

Prof Robert Stewart, MD

Department of Psychological Medicine, Institute of Psychiatry, Psychology & Neuroscience, King's College London, London, UK.

South London and Maudsley NHS Foundation Trust, London, UK

Dr Sumithra Velupillai, PhD

Department of Psychological Medicine, Institute of Psychiatry, Psychology & Neuroscience, King's College London, London, UK.

For peer review only

Dr Johnny Downs, PhD

Department of Child and Adolescent Psychiatry, King's College London, Institute of Psychiatry, Psychology & Neuroscience London, UK.

South London and Maudsley NHS Foundation Trust, London, UK

* Authors contributed equally

Corresponding author

Johnny Downs, Department of Child and Adolescent Psychiatry, Institute of Psychiatry, Psychology & Neuroscience, King's College London, Box PO84, 16 De Crespigny Park, London SE5 8AF, UK. johnny.downs@kcl.ac.uk

Word count

3,954

For peer review only - http://bmjopen.bmj.com/site/about/guidelines.xhtml

ABSTRACT

Objectives: Attention Deficit Hyperactivity Disorder (ADHD) is a prevalent childhood disorder, but often goes unrecognised and untreated. To improve access to services, accurate predictions of populations at high risk for ADHD are needed for effective resource allocation. Using a unique linked health and education data resource, we examined how machine learning (ML) approaches can predict risk of ADHD.

Design: Retrospective population cohort study.

Setting: South London (2007-2013).

Participants: n=56,258 pupils with linked education and health data.

Primary outcome measures: Using area under the curve (AUC), we compared the predictive accuracy of four ML models and one Neural Network for ADHD diagnosis. Ethnic group and language biases were weighted using a fair pre-processing algorithm.

Results: Random Forest and Logistic Regression prediction models provided the highest predictive accuracy for ADHD in population samples (AUC 0.86, 0.86 respectively) and clinical samples (AUC 0.72, 0.70). Precision-recall curve analyses were less favourable. Sociodemographic biases were effectively reduced by a fair pre-processing algorithm without loss of accuracy.

Conclusions: ML approaches using linked routinely collected education and health data offer accurate, low cost, and scalable prediction models of ADHD. These approaches could help identify areas of need and inform resource allocation. Introducing 'fairness weighting' attenuates some sociodemographic biases which would otherwise underestimate ADHD risk within minority groups.

Key words: Attention Deficit Disorder with Hyperactivity; Child Psychiatry; Machine Learning; Neural Networks; Algorithms

For peer review only

Strengths and limitations of this study

- We leveraged a large and innovative data linkage between routinely collected health and school data
- Machine learning approaches were applied to predict Attention Deficit Hyperactivity Disorder (ADHD) in a population and clinical cohort
- Models were weighted to account for ethnic group and language biases
- Further validation of these models is needed to establish the stability and generalisability of these models

For peer review only

INTRODUCTION

Attention Deficit Hyperactivity Disorder (ADHD) is a neurodevelopmental disorder characterised by a persistent pattern of inattention, hyperactivity and impulsive behaviour.[1] Although symptoms emerge in pre-school, ADHD tends to lead to clinical levels of impairment during the first few years of statutory education.[2] ADHD can lead to poor outcomes across social, educational and health domains.[3, 4] It can be treated,[5] but substantial diagnostic delays preclude effective early intervention.[6] It is therefore vital that adequate resources are made available for timely ADHD detection, diagnosis and intervention. To effectively allocate adequate resources for ADHD, commissioners need accurate information on the level of need in schools, services and geographic regions.

Pooled estimates from population surveys suggest that 5.3% of the global population meet criteria for ADHD.[2] This could be taken as a broad indicator of level of need in any given population, but some cases identified via population surveys will not be sufficiently severe or complex to require input from clinical services. Therefore, prevalence estimates from population surveys likely over-estimate the true level of need in the population. Alternatively, level of need could be estimated from the prevalence of ADHD cases presenting to clinical services. However, only a fraction of cases requiring input seek help, receive a referral,

For peer review only - http://bmjopen.bmj.com/site/about/guidelines.xhtml

diagnosis and treatment from clinical services.[7] Therefore, prevalence estimates from clinical services likely under-estimate the true level of need in the population.

Overall, there remains a need for accurate indicators of level of need which can inform commissioning and resource-distribution. At the school-level, mental health screening questionnaires administered by teachers can be used to identify pupils at risk of ADHD, which in could turn provide an estimate of ADHD prevalence for that school.[8] However, this approach is resource-intensive, and cannot practically be scaled up to larger populations to estimate burden of need in a wider geographic area.[9] Machine learning techniques may be able to predict ADHD diagnosis at the individual-level, but existing approaches again use data sources which cannot be practically scaled up to larger populations, such as Magnetic Resonance Imaging and electroencephalography data.[10-13] Such models might also be prone to systematic biases[14]: ADHD is under-diagnosed and under-treated among ethnic minority groups, who are therefore unlikely to be adequately represented in the data on which such models are built, unless efforts are made to adjust for such biases.[15, 16]

Therefore, there remains a need for accurate and unbiased ADHD prediction methods which are scalable to large populations or geographic regions. Administrative pupil-level data are already routinely collected and curated by most schools, and are available for whole populations.[17] If machine learning approaches can be effectively applied to these data to build validated and unbiased models predicting childhood ADHD, they could be used to estimate burden of need and guide resource allocation at area-, school- and service-levels. In this study, we applied machine learning approaches to a large, linked administrative school and clinical dataset to: (1) classify pupils in the population with a high likelihood of clinical ADHD, (2) differentiate pupils with ADHD from pupils with other clinical disorders, (3) explore the contribution of demographic, socioeconomic, and education features to an ADHD diagnosis, and (4) evaluate a potential method to reduce sociodemographic biases.

For peer review only - http://bmjopen.bmj.com/site/about/guidelines.xhtml

METHODS

Study design

This was a retrospective cohort study, using an existing data linkage between the National Pupil Database (NPD) and South London and Maudsley NHS Foundation Trust Child and Adolescent Mental Health Services (SLaM CAMHS) (Table S1).[18, 19]

SLaM is one of Europe's largest providers of mental healthcare, serving the London boroughs of Croydon, Lambeth, Lewisham and Southwark. SLaM is the monopoly provider of local CAMHS services, including ADHD diagnostic services.[18] Anonymised individual-level clinical data for these services are accessible for research via the Clinical Record Interactive Search (CRIS).

CRIS data for SLaM CAMHS services have been linked at the individual-level to the NPD. The NPD is a longitudinal database of pupil- and school-level data.[17] The NPD pupil census is collected annually and contains a snapshot of pupils attending state-maintained schools in England, while NPD attainment datasets hold data for all statutory assessments that pupils complete during primary and secondary education.

The CRIS-NPD linkage process has been described in detail elsewhere [20, 21]. Linkage was conducted for referrals to SLaM between 2007 and 2013, for children and adolescents aged 4-18 years. Fuzzy deterministic matching was conducted based on personal identifiers in CRIS, which were sent to the Department for Education for linkage to the NPD. The match rate was 82.5%. As a result of this data linkage, we had access to educational records for pupils who were referred to SLaM from both inside and outside the catchment area, and local pupils who were not referred to SLaM.

Ethics approval

The CRIS data resource, including the linked data here, has received research ethics approval for secondary analyses (Oxford REC C, reference 18/SC/0372). CRIS operates an opt-out system, where all patients have the ability to opt-out of their anonymised data being used for research. At the time of submission, JD had full ongoing access to the data.

For peer review only - http://bmjopen.bmj.com/site/about/guidelines.xhtml

Patient and Public Involvement

The health and education data linkage used in this study was developed in consultation with several clinical, patient and caregiver groups via the SLaM Biomedical Research Centre (BRC) patient engagement programme.[19] A lay summary of the data linkage can be viewed at https://www.maudsleybrc.nihr.ac.uk/facilities/clinical-record-interactive-search-cris/cris-data-linkages/.

Participants

The analysed sample comprised children aged between 6 and 7 years, who were enrolled in mainstream state educational services (not specialist or alternative educational facilities), and who had education and attainment characteristics as captured at the Early Years Foundation Stage (EYFSP; typically aged 4-5 years) and at Key Stage 1 (KS1; typically aged 6-7 years). Pupils were included if they were resident of the SLaM catchment area from 1st September 2007 to 31st September 2013. A resulting n=57,149 pupils were eligible for inclusion.

Main outcome

Individual-level diagnosis of ADHD was measured from both structured and unstructured diagnosis fields in CRIS between September 2007 and August 2013. In structured fields, ADHD diagnosis was defined as International Classification of Diseases 10th Revision (ICD-10) codes F90.0, F90.1, F90.8, or F90.9 (Hyperkinetic, other Hyperkinetic Disorders, Attention Deficit Disorder, Hyperkinetic Conduct Disorder).[22] In unstructured fields, ADHD diagnosis was identified and manually evaluated using previously established natural language processing techniques (PPV ~0.82).[23-26]

Educational features and other covariates

School performance indicators were collected from EYFSP and KS1 datasets in the NPD. The EYFSP comprises teacher-assessed social, linguistic, physical and cognitive development at the end of the first year of schooling.[27] KS1 comprises teacher-assessed English, maths and science at the end of the third year of formal education.[28] Gender, age,

For peer review only - http://bmjopen.bmj.com/site/about/guidelines.xhtml

ethnicity, Free School Meal eligibility (eFSM; a proxy measure for deprivation), attendance, first language, Special Educational Need (SEN) and looked after child (LAC) status were also measured from the NPD. In total, 45 characteristics were included (Table S2 and Table S3).

Predictive model preparation and building

To minimise the potential for reverse causality, we excluded pupils who were diagnosed with ADHD prior to their KS1 assessment (n=77; 10.5% of ADHD cases). We used a complete case approach, excluding pupils with missing data from our eligible sample (n=814; 1.4%). The resulting n=56,258 formed our population cohort.

To develop and compare machine learning classifiers, the dataset was randomly divided into a training set (n=42,192; 75.0%), validation set (n=7,033; 12.5%) and test set (n=7,033; 12.5%) with a similar proportion of ADHD cases (~1%) in each set. The training set was used to train the algorithms, while the validation set was used for model refinement (e.g., hyperparameter tuning) and comparison. Final performance was evaluated on the test set. We compared several machine learning algorithms to classic statistical Logistic Regression (LR) approaches, and also a supervised approach based on neural networks, using a Multilayer Perceptron (MLP). The following classifiers were compared: LR, Random Forest (RF), Support Vector Machines (SVM), Gaussian Naive Bayes (GNB), and the MLP. For GNB, we first tested that continuous features associated with each class were distributed according to a Gaussian distribution. For the neural network, we used a four-layer architecture (input layer, two hidden layers, output layer).[29]

Selection procedure for machine learning algorithms

Model performances were measured with the area under the receiver operator characteristics curve (AUC). We also report precision, recall and F1-score with a fixed classification threshold of 0.5. The F1-score was calculated using the "weighted" option, which calculates metrics for each label and finds their average weighted by support. For each given value, a 95% confidence interval (CI) of the outcome measure was estimated by bootstrapping 100 times the subjects in the set. We also examined the precision-recall curve (or PR Curve) with precision (y-axis) and recall (x-axis) plotted for different probability thresholds. All the experiments with machine learning algorithms were conducted in Python with functions from

For peer review only - http://bmjopen.bmj.com/site/about/guidelines.xhtml

For peer review only

the scikit learn library for classification (version 0.20.3).[30] The MLP was implemented using the Keras framework (version 2.2.4).

Tuning, resampling and ensemble methods for classification

For LR and RF, tuning of the hyperparameters was made using grid search with 10-fold cross validation on the validation set (full details available by request to the author). The GNB classifier had no parameters to tune. Tuning of SVM was done empirically (Table S4). To compensate for class imbalance of ADHD outcomes, we compared two different methods: random subsampling and Tomek Links.[31] For random subsampling, we successively fitted our model to multiple sub-samples. Each sample contained all ADHD cases and 1000 subjects without any ADHD diagnosis. We calculated the AUC for each fitted model and took the average value of AUC. For Tomek links, we used two approaches – removing all links from the dataset and removing the majority class samples that were part of a Tomek link. In an attempt to improve classification performance, ensemble methods were used on selected machine learning methods.

Selection and testing the final models

Two criteria were defined for the final models' selection: performance and transparency, i.e. the models' ability to determine feature contribution, which enabled examination of potential biases inherent to the data resources used. High performing models were considered *a priori* as having AUC ≥0.85 on the validation set.

Models which were both transparent and performed well were tested for diagnostic efficiency, the purpose being to assess whether the predictive performance from the population sample could generalize to clinical populations. To test the specificity of the best models for ADHD from other clinical mental health problems, we repeated our approach on a purely clinical cohort containing only the children who were present in the CRIS dataset, thus containing samples of children who presented with ADHD and non-ADHD diagnoses. Once all models were tested on the full set of features, we then refined the models to improve accuracy, reduce overfitting and increase potential for translation into busy clinical settings – making the model less vulnerable to missing information. To identify the most important features for LR models we used Beta coefficients, while RF feature importance represents the

For peer review only - http://bmjopen.bmj.com/site/about/guidelines.xhtml

decrease in node impurity on a given branch within a decision tree, weighted by the probability of reaching that node.

Bias reduction

We investigated bias reduction for the LR and RF models trained on the population cohort using the AI Fairness 360 toolkit.[32] We used a pre-processing algorithm as we could access and modify the data directly and the data was inherently biased due to unfair clinical case ascertainment. Pre-processing algorithms learn a new representation based on protected attributes, i.e. features that partition the data set into groups that should have equal probability of getting a favourable label.[33] In our case, protected attributes were represented by ethnicity and language status, while the favourable label is an ADHD diagnosis. The Reweighing algorithm was chosen for its ability to treat two protected attributes at the same time and for the transparency of its fairness process.[34] This algorithm modified the dataset by generating different weights for the training examples in each (group, label) combination, to ensure fairness before classification.

Figure S1 displays the pre-processing fairness pipeline. We defined White and English speaking as the 'privileged' group, and non-White and non-English speaking as the 'unprivileged' group, thus obtaining two non-overlapping groups to be weighted differently. Other combinations (non-White and English speaking, White and non-English speaking) were not explicitly reweighted.

For peer review only - http://bmjopen.bmj.com/site/about/guidelines.xhtml

RESULTS

Cohort characteristics

In total, n=652 pupils in our sample were diagnosed with ADHD (1.16% of the population cohort; 15.6% of the clinical cohort) (Table 1). In the population cohort, gender was evenly balanced, and pupils were predominantly black (n=22,904, 40.71%) and spoke English as a first language (n=37,432, 66.54%). However, males were over-represented in the clinical cohort (n=2848, 68.17%), as were white ethnic groups (n=1712, 40.98%) and English first language speakers (n=3390, 81.14%). This is consistent with national surveys which report greater psychiatric morbidity among males aged 5 to 10 years, and higher rates of help-seeking and detection among white, English speaking groups.[35, 36] Compared to the population cohort, the clinical cohort also had a lower EYFSP and KS1 attainment, lower attendance, and higher rates of exclusion, eFSM, LAC and SEN.

For peer review only - http://bmjopen.bmj.com/site/about/guidelines.xhtml

For peer review only

Table 1: Characteristics of the population cohort and clinical cohort

	Population cohort n=56258		Clinical cohort n=4178	
ADHD (n, %)	652	1.16%	652	15.61%
Gender (male) (n, %)	28434	50.54%	2848	68.17%
Summer birth (n, %)	19274	34.26%	1443	34.53%
English as first language (n, %)	37432	66.54%	3390	81.14%
Ethnicity (n, %)				
Asian	4833	8.59%	129	3.09%
Black	22904	40.71%	1461	34.97%
Chinese	484	0.86%	10	0.24%
White	18218	32.38%	1712	40.98%
Mixed	7116	12.65%	667	15.97%
Other	2702	4.80%	189	4.52%
EYFSP attainment (range; mean,(SD))				
Personal, social, & emotional development	0.28 – 6.60	4.98 (0.99)	0.28 – 6.60	4.32 (1.20)
Communication, language & literacy	1.31 – 6.67	4.97 (0.99)	1.31 – 6.67	4.39 (1.16)
Problem solving, reasoning & numeracy	0.86 – 6.54	4.98 (0.99)	0.86 – 6.54	4.46 (1.27)
Knowledge & understanding of the world	0.95 – 6.58	4.97 (0.99)	0.95 – 6.58	4.54 (1.23)
Physical development	-0.01 – 6.43	4.98 (0.99)	-0.01 – 6.43	4.45 (1.31)
Creative development	0.58 – 6.76	4.97 (0.99)	0.58 – 6.76	4.50 (1.25)
KS1 attainment (range; mean, (SD))				
Maths	0 – 27	15.50 (4.10)	3 – 27	13.24 (4.57)
Reading	0 – 27	15.66 (3.88)	3 – 27	13.66 (5.02)
Writing	0 – 27	14.31 (3.71)	3 – 27	11.61 (4.58)
Science	0 – 27	15.23 (3.55)	3 – 21	13.29 (4.43)

	EYFSP		KS1		EYFSP		KS1	
	n	%	n	%	n	%	n	%
eFSM (n, %)	18175	32.31%	18177	32.31%	1880	44.50 %	1882	45.05%
Looked after child status (n, %)	98	0.17%	202	0.36%	<10	<10	103	2.47%
Mainstream schooling (n, %)	-	-	55854	99.28%	-	-	4006	95.88%
Exclusion (n, %)	-	-	690	1.23 %	-	-	494	11.82%
No SEN (n, %)	45113	80.19%	45116	80.20%	2031	48.61%	2031	48.61%
Statement (n, %)	924	1.64%	922	1.64%	356	8.52%	356	8.52%
SEN school action (n, %)	5766	10.25%	5766	10.25%	675	16.16%	675	16.16%
SEN school action plus (n, %)	4454	7.92%	4453	7.92%	1116	26.71%	1116	26.71%
SEN Behavioural, social & emotional (n, %)	331	0.6%	331	0.6%	<10	<10	<10	<10

For peer review only

Model performance: identifying ADHD from population cohort

Once all models were tested on the full set of 68 features, we refined the models to improve accuracy, reduce overfitting and increase potential for translation into busy clinical settings. Table 2 displays AUC, Precision, Recall and F1-score, including confidence parameters for all tuned models on both validation and test sets, for classifying ADHD from the population cohort (Figure 1 for ROC analysis). Hyperparameter tuning improved RF significantly. Precision was high (above 0.985 on both the validation and test set) for LR, RF and GNB. LR, RF and the MLP all had an AUC≥0.85 on both the validation and the test set. SVM did not discriminate ADHD diagnosis as well (AUC<0.82). Overall, LR and RF were the highest performing and transparent models.

Table 2: Comparison of models: (i) Predictive performance of all trained models on the population cohort (ii) Performance of LR and RF in identifying ADHD from other child-onset psychiatric disorders within the clinical cohort. Median and 95% Confidence Interval.

**Models** [a]	**AUC** [b]		**Precision**		**Recall**		**F1-score**	
	**Validation**[c,d]	**Test**[c,d]	**Validation**[c,d]	**Test**[c,d]	**Validation**[c,d]	**Test**[c,d]	**Validation**[c,d]	**Test**[c,d]
**Population cohort**								
**LR**	0.862 [0.840-0.874]	0.900	0.985 [0.985-0.986]	0.987	0.835 [0.822- 0.848]	0.827	0.900 [0.892- 0.908]	0.895
**RF**	0.857 [0.842-0.869]	0.860	0.985 [0.985-0.986]	0.986	0.816 [0.802- 0.833]	0.802	0.889 [0.880-0.899]	0.880
**SVM**	0.811 [0.793-0.833]	0.830	0.980 [0.979-0.981]	0.981	0.936 [0.930-0.943]	0.908	0.957 [0.954- 0.96]	0.942
**GNB**	0.855 [0.846-0.873]	0.849	0.985 [0.985-0.986]	0.987	0.835 [0.822-0.850]	0.707	0.890 [0.882-0.909]	0.817
**MLP**	0.855 [0.834-0.864]	0.883	0.978 [0.975-0.987]	0.977	0.988 [0.982-0.990]	0.988	0.983 [0.979- 0.985]	0.983
**Clinical cohort**								
**LR**	0.718 [0.701-0.735]	0.694	0.813 [0.804-0.834]	0.797	0.659 [0.647-0.673]	0.646	0.705 [0.694-0.717]	0.693
**RF**	0.699 [0.682-0.71]	0.689	0.808 [0.784-0.823]	0.808	0.639 [0.613-0.672]	0.600	0.689 [0.665-0.715]	0.654

[a] Logistic Regression (LR), Random Forest (RF), Support Vector Machines (SVM), Gaussian Naive Bayes (GNB), Multilayer Perceptron (MLP)

[b] Area Under Curve (AUC)

[c] ADHD cases: n=82

[d] Non-ADHD cases: n=6951 in population cohort, n=441 in clinical cohort

[Figure 1: ROC curves for the tuned models]

For peer review only - http://bmjopen.bmj.com/site/about/guidelines.xhtml

Model performance: identifying ADHD from clinical cohort

Table 2 displays Precision, Recall, F1-score and final hyperparameters trained, validated and tested on the clinical cohort. Precision was ~0.80 for ADHD on both the validation and test set for both models on the clinical cohort. Both models' overall predictive performance (AUC 0.70) was much lower relative to their performance on the population cohort.

Resampling methods used to deal with class imbalance did not improve the performances significantly. Overall, Random Subsampling performed better than Tomek Links. As for ensemble approaches, a slight improvement in performance was noticed for RF only, while LR obtained better performance when using resampling methods. The precision-recall curves (PRC) for both population and clinical cohorts are displayed in Figure S2 and Figure S3. The models show significantly lower performance than ROC curve results, although they do demonstrate greater performance than a random classifier, based on PRC metrics as summarised in Table S5 comparing the PRC and ROC for LR, RF and random classification.

Bias reduction using English language and white ethnicity as protective attributes

Table 3 displays results on the population cohort (test set) when using the LR and RF models trained on transformed data (i.e., with instance weights from the fairness Reweighing algorithm). The Disparate Impact (DI) score displays the ratio of the probability of a favourable outcome for unprivileged instances and the probability of a favourable outcome for privileged instances (close to 1 in a fair dataset). The reweighing algorithm obtained a considerable improvement of the disparate impact (from 0.15-0.60 to 1), without harming the models' performance on ADHD classification: for the LR/RF models the AUC remained above 0.880/0.858 in all cases.

Table 3. Model performance on the reweighed fair dataset resulting from the bias reduction algorithm.

Models [a]	AUC	Precision	Recall	F1-score	DI before	DI after
LR[b]	0.880	0.986	0.810	0.885	0.149	~1.000
RF[b]	0.858	0.986	0.797	0.876	0.149	~1.000

[a] Logistic Regression (LR), Random Forest (RF), Disparate Impact (DI)

[b] Re-weighting model based on the 'privileged' group being both White and speaking English as a first language

For peer review only - http://bmjopen.bmj.com/site/about/guidelines.xhtml

Feature contribution for ADHD prediction in population and clinical cohorts

Table 4 displays the most important features overall, plus ethnicity, for LR and RF models in the population and clinical cohorts (all features in Table S6). KS1 writing performance was a strongly protective/discriminant factor against ADHD. Gender, KS1 attendance, SEN status, and personal, social and emotional development were also strongly discriminant features, while the importance of English language and ethnicity was reduced following reweighing.

For peer review only

For peer review only - http://bmjopen.bmj.com/site/about/guidelines.xhtml

Table 4. Feature contribution for ADHD prediction and the impact of fairness re-weighting.

	**Population cohort (n= 56257)**				**Clinical cohort (n= 4178)**	
	**LR**		**RF**		**LR**	**RF**
	**unweighted**	**weighted**	**unweighted**	**weighted**		
**KS1 Writing score**	**-0.761**	**-0.858**	**0.106**	**0.100**	**-0.407**	**0.109**
**EYFSP Personal, Social and Emotional Development**	**-0.841**	**-1.000**	**0.051**	**0.065**	**-0.314**	0.047
**KS1 Attendance (%)**	**0.958**	**1.104**	0.010	**0.008**	0.093	**0.059**
**Male gender**	0.608	**0.626**	0.029	**0.061**	**0.389**	**0.142**
**KS1 No SEN**	-0.198	-0.169	**0.146**	**0.138**	-0.094	**0.087**
**English as first language**	**0.696**	**0.183**	0.049	**0.006**	0.113	0.032
**EYFSP Attendance (%)**	**-0.983**	**-1.019**	0.010	0.005	0.093	0.050
**EYFSP Problem Solving, Reasoning and Numeracy**	**0.201**	**0.318**	0.008	**0.005**	**0.183**	0.019
**White ethnicity**	**0.224**	0.022	0.004	<0.001	0.078	0.006

Note: For the population cohort, coefficients of the model when trained on the Reweighed dataset are displayed. Feature importance metrics are different for LR and RF, and not directly comparable. LR Beta coefficients represent the log-odds of a given feature, negative values correspond with a decrease in the probability of the case being classified as ADHD and vice versa. RF feature importance is calculated as the decrease in node impurity on a given branch within a decision tree, weighted by the probability of reaching that node. The feature importance value presented represents the average feature importance over all the trees. The table displays the top 5 features for at least one model (marked in bold for the corresponding column) and top 20 features for all 4 models. The feature "White ethnicity" was added for comparing its significance after reducing bias. Ranking was made by decreasing significance. For the population cohort, coefficients of both models when trained on the Reweighed dataset using both English and White as protected attributes, are displayed.

For peer review only

For peer review only - http://bmjopen.bmj.com/site/about/guidelines.xhtml

DISCUSSION

This study aimed to predict pupils with a high likelihood of ADHD diagnosis using data available at the population-level. To our knowledge, it is the first study to apply machine learning approaches to large scale, routinely collected linked health and education data. These innovative methods show that ADHD in general population and clinical cohorts can be predicted with high levels of accuracy. Promisingly, this level of predictive accuracy is comparable to traditional survey-based screening methods in ADHD, and to other studies using routinely collected data to predict health outcomes.[37-40]

These findings suggest that machine learning approaches can accurately predict ADHD in large general population and clinical cohorts using existing and widely available administrative data. These models may therefore be suited to provide health intelligence to local policy makers at low cost, and inform decisions on the allocation of disorder-specific resources at an area-, school- or service-level. Tentatively, these models may also offer a means to predict ADHD at the individual-level, although greater precision would be desirable for this purpose – this is an area for future work.

We also demonstrate that sociodemographic biases inherent in the data and algorithms can be reduced with a fairness reweighing step to ensure that pupils from certain backgrounds are not systematically overlooked as being at risk of ADHD. In a geographic area with a large proportion of people from ethnic minority and non-English speaking backgrounds, minimising such biases is crucial for accurately predicting need for ADHD provision in the population.[26]

This therefore highlights the need to build fairness measures which weight for limited access to diagnosis in certain ethnic and social groups. However, it is important to note that these statistical approaches are unlikely to remove all potential biases inherent to service provision data: for example, parents also influence help-seeking for ADHD, but their characteristics are not fully captured in datasets like the NPD, and so could not be used in reweighing processes.[41] Subset scanning could be a means to detect biases in certain subgroups.[34] It should also be noted that the reweighing algorithm we used was based on the assumption that different ethnic and language groups should have equal probability of a favourable label (i.e. ADHD): under these parameters, Optimized Preprocessing could also have been applied, as it

For peer review only - http://bmjopen.bmj.com/site/about/guidelines.xhtml

ensures both group fairness and individual fairness, where similar individuals are treated similarly.[42] Further work could explore these methods.

Strengths and limitations

Our study benefited from a large and novel data linkage capturing almost all pupils from a defined geographic catchment. This reduces the risk of building models on biased and non-representative samples. These approaches are also scalable, as they use information already routinely collected by schools. We used a set of machine learning algorithms that are state-of-the-art techniques and are representative of the wide spectrum of machine learning methods: an ensemble method (RF), a kernel-based method (SVM), a Bayesian method (Naive Bayes), and a neural network (MLP).

However, some limitations should be highlighted. While we sought to address biases in clinical data using reweighing algorithms, biases in the education data (such as inconsistent representation of private or home-schooled pupils) have not been addressed. We also handled missing data using a complete-case analysis, which can introduce bias if the missing data are not randomly distributed. An alternative would have been to explore imputation methods; however, given that we compare models of varying complexities (from Logistic Regression to MLPs), we would have had to adapt the imputation method to the model, resulting in various datasets. The comparison between the resulting models would therefore have been unfair. Encouragingly, the proportion of missing data was very low, and therefore unlikely to materially bias the findings. Nonetheless, further validation of these models is warranted.[43]

Our hypothesis in this paper is that diagnostic and treatment discrepancies between different ethnicities and language groups are due to disparity. It could be argued that such discrepancies are in fact reflective of genuine underlying differences in ADHD prevalence in these populations. However, existing evidence suggests that ADHD is under-diagnosed and under-treated in certain ethnic groups; plausible mechanisms along the care-seeking pathway which may result in these disparities have been discussed elsewhere.[15, 16]

For re-weighing, we dichotomised ethnicity as White/non-White, and language as English/non-English– conducting a more granular re-weighing procedure using more precise groupings would be an important area for future research. We also did not conduct re-

For peer review only - http://bmjopen.bmj.com/site/about/guidelines.xhtml

weighing on the basis of any other sociodemographic characteristics such as gender or deprivation, and make no assumptions as to their role in differential diagnoses – again, these could be areas for future work.

To identify ADHD cases, we used diagnostic codes recorded in structured fields. Evidence suggests that ADHD can be classified from diagnostic codes in electronic health records with a high degree of accuracy; however, such methods of ascertainment are imperfect, and the possibility of diagnostic or administrative misclassification does remain.[44, 45]

Replication in other regions or populations would also be important to assess the generalisability of these models, although at present, other educational and clinical data linkages on this scale are scarce. Finally, some of the features used in the analysis had very few observations for the ADHD cases. The potential impact of this is understudied, and warrants further investigation.[46, 47]

Conclusions

Overall, this study demonstrates that machine learning approaches using readily available education and clinical data show promise in predicting ADHD in source populations. It also highlights that biases inherent in routinely-collected data can be mitigated using fair weighting processes. With further validation and replication work, these methods have the potential to estimate burden of need for ADHD provision, thereby informing resource allocation and policy decisions.

Funding statement

JD is supported by NIHR Clinician Science Fellowship award (CS-2018-18-ST2-014) and has received support from a Medical Research Council Clinical Research Training Fellowship (MR/L017105/1) and Psychiatry Research Trust Peggy Pollak Research Fellowship in Developmental Psychiatry. SV was additionally supported by the Swedish Research Council (2015-00359), Marie Skłodowska Curie Actions, Cofund. RS, LC, JD and SV were additionally supported by a Medical Research Council Mental Health Data Pathfinder Award to King's College London. The Clinical Record Interactive Search (CRIS),

For peer review only - http://bmjopen.bmj.com/site/about/guidelines.xhtml

RS, JD, NV and SV are part-supported by the National Institute of Health Research Biomedical Research Centre at the South London and Maudsley NHS Foundation Trust and King's College London. RS is additionally part funded by the National Institute for Health Research (NIHR) Applied Research Collaboration South London (NIHR ARC South London) at King's College Hospital NHS Foundation Trust, and the DATAMIND HDR UK Mental Health Data Hub (MRC grant MR/W014386). AW is in receipt of a PhD studentship funded by the National Institute for Health Research (NIHR) Biomedical Research Centre at South London and Maudsley NHS Foundation Trust and King's College London. The views expressed are those of the author and not necessarily those of the NHS, the NIHR or the Department of Health and Social Care. The authors are very grateful to Richard White and members of DfE National Pupil Database team, who provided invaluable support throughout the project.

The funder of the study had no role in study design, data collection, data analysis, data interpretation, or writing of the report. The corresponding author had full access to all the data in the study and had final responsibility for the decision to submit for publication.

Competing interests statement

The authors report no potential conflicts of interest.

Contribution statement

The study was conceived by LTM, NV and JD. Data extraction was carried out by JD. Data analysis was undertaken by LTM and NV. Reporting of findings was led by LTM and NV with support from JD, AW, LC, SV and RS. All authors contributed to manuscript preparation and approved the final version.

For peer review only - http://bmjopen.bmj.com/site/about/guidelines.xhtml

Data sharing statement

Data can be accessed with permissions from both the Department for Education and South London and Maudsley NHS Foundation Trust.

REFERENCES

1. Rutter M. *Multiaxial classification of child and adolescent psychiatric disorders: the ICD-10 classification of mental and behavioural disorders in children and adolescents*. Cambridge University Press; 1996.
2. Polanczyk G, De Lima MS, Horta BL, Biederman J, Rohde LA. The worldwide prevalence of ADHD: a systematic review and metaregression analysis. *American journal of psychiatry*. 2007;164(6):942-948.
3. Fleming M, Fitton CA, Steiner MFC, et al. Educational and Health Outcomes of Children Treated for Attention-Deficit/Hyperactivity Disorder. *JAMA Pediatr*. Jul 3 2017;171(7):e170691. doi:10.1001/jamapediatrics.2017.0691
4. Wehmeier PM, Schacht A, Barkley RA. Social and emotional impairment in children and adolescents with ADHD and the impact on quality of life. *J Adolesc Health*. Mar 2010;46(3):209-17. doi:10.1016/j.jadohealth.2009.09.009
5. Cortese S, Adamo N, Del Giovane C, et al. Comparative efficacy and tolerability of medications for attention-deficit hyperactivity disorder in children, adolescents, and adults: a systematic review and network meta-analysis. *Lancet Psychiatry*. Sep 2018;5(9):727-738. doi:10.1016/S2215-0366(18)30269-4
6. Fridman M, Banaschewski T, Sikirica V, Quintero J, Chen KS. Access to diagnosis, treatment, and supportive services among pharmacotherapy-treated children/adolescents with ADHD in Europe: data from the Caregiver Perspective on Pediatric ADHD survey. *Neuropsychiatr Dis Treat*. 2017;13:947-958. doi:10.2147/NDT.S128752
7. Bussing R, Zima BT, Gary FA, Garvan CW. Barriers to detection, help-seeking, and service use for children with ADHD symptoms. *The journal of behavioral health services & research*. 2003;30(2):176-189.
8. Fazel M, Hoagwood K, Stephan S, Ford T. Mental health interventions in schools 1: Mental health interventions in schools in high-income countries. *Lancet Psychiatry*. Oct 2014;1(5):377-387. doi:10.1016/S2215-0366(14)70312-8
9. Weist MD, Rubin M, Moore E, Adelsheim S, Wrobel G. Mental health screening in schools. *J Sch Health*. Feb 2007;77(2):53-8. doi:10.1111/j.1746-1561.2007.00167.x
10. Heller MD, Roots K, Srivastava S, Schumann J, Srivastava J, Hale TS. A Machine Learning-Based Analysis of Game Data for Attention Deficit Hyperactivity Disorder Assessment. *Games Health J*. Oct 2013;2(5):291-8. doi:10.1089/g4h.2013.0058
11. Sen B, Borle NC, Greiner R, Brown MRG. A general prediction model for the detection of ADHD and Autism using structural and functional MRI. *PLoS One*. 2018;13(4):e0194856. doi:10.1371/journal.pone.0194856
12. Sun H, Chen Y, Huang Q, et al. Psychoradiologic Utility of MR Imaging for Diagnosis of Attention Deficit Hyperactivity Disorder: A Radiomics Analysis. *Radiology*. May 2018;287(2):620-630. doi:10.1148/radiol.2017170226
13. Tenev A, Markovska-Simoska S, Kocarev L, Pop-Jordanov J, Muller A, Candrian G. Machine learning approach for classification of ADHD adults. *Int J Psychophysiol*. Jul 2014;93(1):162-6. doi:10.1016/j.ijpsycho.2013.01.008

For peer review only - http://bmjopen.bmj.com/site/about/guidelines.xhtml

14. Rajkomar A, Hardt M, Howell MD, Corrado G, Chin MH. Ensuring Fairness in Machine Learning to Advance Health Equity. *Ann Intern Med*. Dec 18 2018;169(12):866-872. doi:10.7326/M18-1990
15. Coker TR, Elliott MN, Toomey SL, et al. Racial and Ethnic Disparities in ADHD Diagnosis and Treatment. *Pediatrics*. Sep 2016;138(3)doi:10.1542/peds.2016-0407
16. Shi Y, Hunter Guevara LR, Dykhoff HJ, et al. Racial Disparities in Diagnosis of Attention-Deficit/Hyperactivity Disorder in a US National Birth Cohort. *JAMA Netw Open*. Mar 1 2021;4(3):e210321. doi:10.1001/jamanetworkopen.2021.0321
17. Jay MA, McGrath-Lone L, Gilbert R. Data Resource: the National Pupil Database (NPD). *Int J Popul Data Sci*. Mar 20 2019;4(1):1101. doi:10.23889/ijpds.v4i1.1101
18. Downs J, Gilbert R, Hayes RD, Hotopf M, Ford T. Linking health and education data to plan and evaluate services for children. *Arch Dis Child*. Jul 2017;102(7):599-602. doi:10.1136/archdischild-2016-311656
19. Downs JM, Ford T, Stewart R, et al. An approach to linking education, social care and electronic health records for children and young people in South London: a linkage study of child and adolescent mental health service data. *BMJ Open*. Jan 29 2019;9(1):e024355. doi:10.1136/bmjopen-2018-024355
20. Downs J, Gilbert R, Hayes RD, Hotopf M, Ford T. Linking health and education data to plan and evaluate services for children. *Archives of Disease in Childhood*. 2017;102(7):599-602. doi:10.1136/archdischild-2016-311656
21. Downs J, Ford T, Stewart R, et al. An approach to linking education, social care and electronic health records for children and young people in South London: a linkage study of child and adolescent mental health service data. *BMJ Open*. 2019;9(1):e024355. doi:10.1136/bmjopen-2018-024355
22. World Health Organization. *International Statistical Classification of Diseases and Related Health Problems*. 10th Revision ed. 2000.
23. Downs J, Hotopf M, Ford T, et al. Clinical predictors of antipsychotic use in children and adolescents with autism spectrum disorders: a historical open cohort study using electronic health records. *Eur Child Adolesc Psychiatry*. Jun 2016;25(6):649-58. doi:10.1007/s00787-015-0780-7
24. Jackson RG, Patel R, Jayatilleke N, et al. Natural language processing to extract symptoms of severe mental illness from clinical text: the Clinical Record Interactive Search Comprehensive Data Extraction (CRIS-CODE) project. *BMJ Open*. Jan 17 2017;7(1):e012012. doi:10.1136/bmjopen-2016-012012
25. O'Connor C, Downs J, McNicholas F, Cross L, Shetty H. Documenting diagnosis in child and adolescent mental healthcare: A content analysis of diagnostic statements in a psychiatric case register. *Children and Youth Services Review*. 2020;113:104948.
26. Perera G, Broadbent M, Callard F, et al. Cohort profile of the South London and Maudsley NHS Foundation Trust Biomedical Research Centre (SLaM BRC) Case Register: current status and recent enhancement of an Electronic Mental Health Record-derived data resource. *BMJ Open*. Mar 1 2016;6(3):e008721. doi:10.1136/bmjopen-2015-008721
27. Early years foundation stage profile handbook. . GOV.UK. Accessed 15 Sep 2019, https://www.gov.uk/government/publications/early-years-foundation-stage-profile-handbook
28. Key stage 1 teacher assessment guidance. GOV.UK. Accessed 15 Sep 2019, https://www.gov.uk/government/publications/key-stage-1-teacher-assessment-guidance
29. Bebis G, Georgiopoulos M. Feed-forward neural networks. *IEEE Potentials*. 1994;13(4):27-31.
30. scikit-learn: machine learning in Python — scikit-learn 0.21.3 documentation. Accessed 15 Sep 2019, https://scikit-learn.org/stable/

For peer review only - http://bmjopen.bmj.com/site/about/guidelines.xhtml

31. Two Modifications of CNN. *IEEE Transactions on Systems, Man, and Cybernetics*. 1976;SMC-6(11):769-772. doi:10.1109/TSMC.1976.4309452
32. Bellamy RK, Dey K, Hind M, et al. AI Fairness 360: An extensible toolkit for detecting and mitigating algorithmic bias. *IBM Journal of Research and Development*. 2019;63(4/5):4: 1-4: 15.
33. d'Alessandro B, O'Neil C, LaGatta T. Conscientious Classification: A Data Scientist's Guide to Discrimination-Aware Classification. *Big Data*. Jun 2017;5(2):120-134. doi:10.1089/big.2016.0048
34. Zhang Z, Neill DB. Identifying significant predictive bias in classifiers. *arXiv preprint arXiv:161108292*. 2016;
35. Ford T, Goodman R, Meltzer H. The British Child and Adolescent Mental Health Survey 1999: the prevalence of DSM-IV disorders. *J Am Acad Child Adolesc Psychiatry*. Oct 2003;42(10):1203-11. doi:10.1097/00004583-200310000-00011
36. Ford T, Hamilton H, Meltzer H, Goodman R. Predictors of Service Use for Mental Health Problems Among British Schoolchildren. *Child Adolesc Ment Health*. Feb 2008;13(1):32-40. doi:10.1111/j.1475-3588.2007.00449.x
37. Algorta GP, Dodd AL, Stringaris A, Youngstrom EA. Diagnostic efficiency of the SDQ for parents to identify ADHD in the UK: a ROC analysis. *Eur Child Adolesc Psychiatry*. Sep 2016;25(9):949-57. doi:10.1007/s00787-015-0815-0
38. Basu S, Narayanaswamy R. A Prediction Model for Uncontrolled Type 2 Diabetes Mellitus Incorporating Area-level Social Determinants of Health. *Med Care*. Aug 2019;57(8):592-600. doi:10.1097/MLR.0000000000001147
39. Hammond R, Athanasiadou R, Curado S, et al. Predicting childhood obesity using electronic health records and publicly available data. *PLoS One*. 2019;14(4):e0215571. doi:10.1371/journal.pone.0215571
40. Xiao C, Choi E, Sun J. Opportunities and challenges in developing deep learning models using electronic health records data: a systematic review. *J Am Med Inform Assoc*. Oct 1 2018;25(10):1419-1428. doi:10.1093/jamia/ocy068
41. Sayal K, Prasad V, Daley D, Ford T, Coghill D. ADHD in children and young people: prevalence, care pathways, and service provision. *The Lancet Psychiatry*. 2018;5(2):175-186.
42. Neill DB. Expectation-based scan statistics for monitoring spatial time series data. *International Journal of Forecasting*. 2009;25(3):498-517.
43. Riley RD, Ensor J, Snell KI, et al. External validation of clinical prediction models using big datasets from e-health records or IPD meta-analysis: opportunities and challenges. *BMJ*. Jun 22 2016;353:i3140. doi:10.1136/bmj.i3140
44. Daley MF, Newton DA, DeBar L, et al. Accuracy of Electronic Health Record–Derived Data for the Identification of Incident ADHD. *Journal of Attention Disorders*. 2017;21(5):416-425.
45. Gruschow SM, Yerys BE, Power TJ, Durbin DR, Curry AE. Validation of the Use of Electronic Health Records for Classification of ADHD Status. *J Atten Disord*. Nov 1 2019;23(13):1647-1655. doi:10.1177/1087054716672337
46. Johnson JM, Khoshgoftaar TM. Survey on deep learning with class imbalance. *Journal of Big Data*. 2019/03/19 2019;6(1):27. doi:10.1186/s40537-019-0192-5
47. van der Ploeg T, Austin PC, Steyerberg EW. Modern modelling techniques are data hungry: a simulation study for predicting dichotomous endpoints. *BMC Med Res Methodol*. Dec 22 2014;14:137. doi:10.1186/1471-2288-14-137

For peer review only - http://bmjopen.bmj.com/site/about/guidelines.xhtml

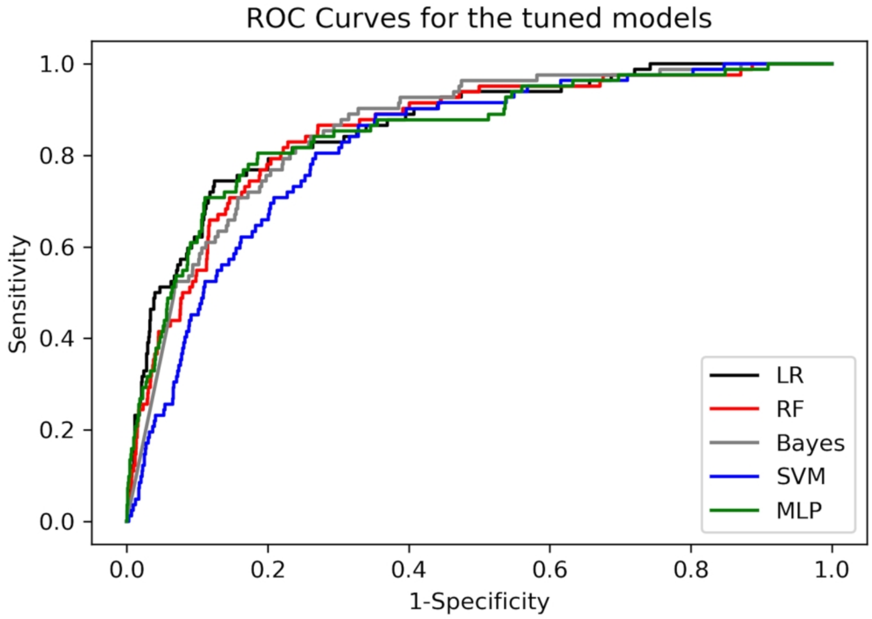

Figure 1: ROC curves for the tuned models

152x101mm (300 x 300 DPI)

Table S1: The RECORD statement – checklist of items, extended from the STROBE statement, that should be reported in observational studies using routinely collected health data. Items specific to cross-sectional/case-control studies have been removed.

	Item No.	STROBE items	Location in manuscript where items are reported	RECORD items	Location in manuscript where items are reported
**Title and abstract**					
	1	(a) Indicate the study's design with a commonly used term in the title or the abstract (b) Provide in the abstract an informative and balanced summary of what was done and what was found	3	RECORD 1.1: The type of data used should be specified in the title or abstract. When possible, the name of the databases used should be included.<br><br>RECORD 1.2: If applicable, the geographic region and timeframe within which the study took place should be reported in the title or abstract.<br><br>RECORD 1.3: If linkage between databases was conducted for the study, this should be clearly stated in the title or abstract.	3
**Introduction**					
Background rationale	2	Explain the scientific background and rationale for the investigation being reported	4-5	N/A	
Objectives	3	State specific objectives, including any prespecified hypotheses	5	N/A	
**Methods**					

For peer review only

Study Design	4	Present key elements of study design early in the paper	5-6	N/A	
Setting	5	Describe the setting, locations, and relevant dates, including periods of recruitment, exposure, follow-up, and data collection	3, 5-6	N/A	
Participants	6	*(a) Cohort study* - Give the eligibility criteria, and the sources and methods of selection of participants. Describe methods of follow-up<br><br>*(b) Cohort study* - For matched studies, give matching criteria and number of exposed and unexposed	6	RECORD 6.1: The methods of study population selection (such as codes or algorithms used to identify subjects) should be listed in detail. If this is not possible, an explanation should be provided.<br><br>RECORD 6.2: Any validation studies of the codes or algorithms used to select the population should be referenced. If validation was conducted for this study and not published elsewhere, detailed methods and results should be provided.<br><br>RECORD 6.3: If the study involved linkage of databases, consider use of a flow diagram or other graphical display to demonstrate the data linkage process, including the number of individuals with linked data at each stage.	6
Variables	7	Clearly define all outcomes, exposures, predictors, potential confounders, and effect modifiers. Give diagnostic criteria, if applicable.	7-8	RECORD 7.1: A complete list of codes and algorithms used to classify exposures, outcomes, confounders, and effect modifiers should be provided. If these cannot be reported, an explanation should be provided.	7-8
Data sources/ measurement	8	For each variable of interest, give sources of data and details of methods of assessment (measurement).	7-8	N/A	

For peer review only

		Describe comparability of assessment methods if there is more than one group			
Bias	9	Describe any efforts to address potential sources of bias	10	N/A	
Study size	10	Explain how the study size was arrived at	7	N/A	
Quantitative variables	11	Explain how quantitative variables were handled in the analyses. If applicable, describe which groupings were chosen, and why	7-8	N/A	
Statistical methods	12	(a) Describe all statistical methods, including those used to control for confounding<br>(b) Describe any methods used to examine subgroups and interactions<br>(c) Explain how missing data were addressed<br>(d) *Cohort study* - If applicable, explain how loss to follow-up was addressed<br>(e) Describe any sensitivity analyses	8-10	N/A	
Data access and cleaning methods		N/A		RECORD 12.1: Authors should describe the extent to which the investigators had access to the database population used to create the study population.<br>RECORD 12.2: Authors should provide information on the data cleaning methods used in the study.	6
Linkage		N/A		RECORD 12.3: State whether the study included person-level, institutional-level, or other data linkage	6

For peer review only

				across two or more databases. The methods of linkage and methods of linkage quality evaluation should be provided.	
**Results**					
Participants	13	(a) Report the numbers of individuals at each stage of the study (*e.g.*, numbers potentially eligible, examined for eligibility, confirmed eligible, included in the study, completing follow-up, and analysed)<br>(b) Give reasons for non-participation at each stage.<br>(c) Consider use of a flow diagram	7	RECORD 13.1: Describe in detail the selection of the persons included in the study (*i.e.,* study population selection) including filtering based on data quality, data availability and linkage. The selection of included persons can be described in the text and/or by means of the study flow diagram.	6-7
Descriptive data	14	(a) Give characteristics of study participants (*e.g.*, demographic, clinical, social) and information on exposures and potential confounders<br>(b) Indicate the number of participants with missing data for each variable of interest<br>(c) *Cohort study* - summarise follow-up time (*e.g.*, average and total amount)	11-12	N/A	
Outcome data	15	*Cohort study* - Report numbers of outcome events or summary measures over time	11-12	N/A	
Main results	16	(a) Give unadjusted estimates and, if applicable, confounder-adjusted estimates and their precision (e.g., 95% confidence interval). Make clear which	N/A	N/A	

For peer review only

		confounders were adjusted for and why they were included<br><br>(b) Report category boundaries when continuous variables were categorized<br><br>(c) If relevant, consider translating estimates of relative risk into absolute risk for a meaningful time period			
Other analyses	17	Report other analyses done—e.g., analyses of subgroups and interactions, and sensitivity analyses	13-16	N/A	
**Discussion**					
Key results	18	Summarise key results with reference to study objectives	17	N/A	
Limitations	19	Discuss limitations of the study, taking into account sources of potential bias or imprecision. Discuss both direction and magnitude of any potential bias	18-19	RECORD 19.1: Discuss the implications of using data that were not created or collected to answer the specific research question(s). Include discussion of misclassification bias, unmeasured confounding, missing data, and changing eligibility over time, as they pertain to the study being reported.	18-19
Interpretation	20	Give a cautious overall interpretation of results considering objectives, limitations, multiplicity of analyses, results from similar studies, and other relevant evidence	17-19	N/A	
Generalisability	21	Discuss the generalisability (external validity) of the study results	19	N/A	
**Other Information**					
Funding	22	Give the source of funding and the role of the funders for the present study and,	19	N/A	

For peer review only

		if applicable, for the original study on which the present article is based			
Accessibility of protocol, raw data, and programming code		N/A		RECORD 22.1: Authors should provide information on how to access any supplemental information such as the study protocol, raw data, or programming code.	21

*Reference: Benchimol, E. I., Smeeth, L., Guttmann, A., Harron, K., Moher, D., Petersen, I., ... & RECORD Working Committee. (2015). The REporting of studies Conducted using Observational Routinely-collected health Data (RECORD) statement. PLoS Med, 12(10), e1001885.

*Checklist is protected under Creative Commons Attribution (CC BY) license.

For peer review only

Table S2: Educational Features

	Feature name	Feature Description
Demographic features	Gender (Male)	Dummy variable indicating male gender.
	Age	Age in years and months at the start of KS1.
	Summer birth	Variable indicating whether the child was born in summer months of school term (1st April- 31st August). These are the youngest children in an academic cohort.
	Ethnicity	Dummy variables indicating ethnic background. We included 6 separate ethnicity dummy variables within the model: Asian, Black, Chinese, White, Mixed, Other.
	Deprivation Indicator (IDACI)	The Income Deprivation Affecting Children Index (IDACI) scores are an indicator of deprivation. The IDACI measures the proportion of all children aged 0 to 15 living in income deprived families. It is a subset of the Income Deprivation Domain which measures the proportion of the population in an area experiencing deprivation relating to low income.
	Academic Year	This feature describes the academic year a child started KS1 (for example 2011).
Additional need indicators	eFSM	Eligibility for Free School Meals is a statutory benefit available to school aged children in England from less affluent backgrounds. eFSM is a commonly used proxy variable for lower socio-economic status.
	Mobility (KS1)	Variable indicating whether a child has moved schools within KS1.
	Mobility (ever)	Variable indicating whether a child has moved schools ever (i.e. during KS1 or the EYFSP).
	Child Looked After Status	Child looked after status indicates that a child has been in the care of their local authority for more than 24 hours.
	English first language	English as a first language refers to whether the child's native language is English or other.
Special Educational Need indicators	No SEN	Dummy variable indicating that the child had no Special Educational Needs.
	SEN School Action	Dummy variable indicating that a child has a school action plan to support their SEN. This might include adapted teaching methods and materials, or in some cases additional adult support. This plan is created by the school.
	SEN School Action+	Dummy variable indicating that a child has had a school action plan to support their SEN, however this has not helped the child make adequate progress. At this point school action plus is required. This involves schools consulting and collaborating with appropriate external support services (for example, a speech and language therapist).
	SEN Statement of needs	Dummy variable indicating that child has a SEN statement of needs. A SEN Statement is a plan set by the Education Authority detailing a child's needs and required support. This is typically made if a child needs support beyond the scope of the school (school action/ school action plus).
	SEN: Autism spectrum disorders	Autism spectrum disorder is a specific SEN. It is a diagnosable disorder that affects social interaction, interests and behaviour.
	SEN: specific learning difficulty	Children with specific learning difficulties, typically struggle with one specific aspect of learning but are able in other areas. For

For peer review only - http://bmjopen.bmj.com/site/about/guidelines.xhtml

		example, they may find reading particularly challenging (but cope well with numbers and letters).
	SEN: moderate learning difficulty	Learning difficulties are formally diagnosed by a clinician. The SEN: moderate learning difficulty code indicates a child's ability to understand and communicate information is moderately impaired.
	SEN: severe learning difficulty	Learning difficulties are formally diagnosed by a clinician. The SEN: severe learning difficulty code indicates a child's ability to understand and communicate information is severely impaired.
	SEN: behavioural, emotional and social difficulties	SEN: Behavioural, social and emotional difficulties is a broad term referring to children who's behaviour poses a barrier to learning. This can be vary in terms of presentation, children may be withdrawn or isolated, disruptive, lack concentration, immature social skills, or other challenging behaviours.
	SEN: speech, language & communication needs	This variable highlights those who find it difficult to listen, understand or communicate with others that adversely affects educational performance.
	SEN: hearing impairment	This variable indicates a child who has either a permanent or fluctuating hearing impairment that adversely affects educational performance.
	SEN: vision impairment	This variable indicates a child who has either a permanent or fluctuating visual impairment that adversely affects educational performance.
	SEN: multi-sensory impairment	This variable indicates a child who has either a permanent or fluctuating multi-sensory impairment (e.g. hearing and vison) that adversely affects educational performance.
	SEN: physical	This variable indicates a child who has either a permanent or fluctuating physical impairment that adversely affects educational performance.
	SEN: other	This variable indicates a child who has a special educational need which is adversely affecting education performance, but not captured in the categories above.
	Mainstream education indicator	Dummy variable indicating that a child is in mainstream education (as opposed to alternative provision).
School attendance and exclusion features	Attendance (%)	The percentage of time within the school year that a child was registered as attending school.
	Exclusion (temporary)	A fixed period exclusion where a child is temporarily moved from school (a maximum of 45 days within a school year).
	Exclusion (permanent)	Permanent exclusion indicating a child has been expelled from school.
School attainment features	EYFSP knowledge of the world	EYFSP sub scale: assesses a child's ability to make sense of their physical world and community on a scale of 1-9 (with 9 being most proficient). This is not a standardised assessment, however it is conducted by a trained professional (Early Years Practitioner) and moderated by the local authority.
	EYFSP problem solving, reasoning and numeracy	EYFSP sub scale: assesses a child's ability to problem solve, reason and perform basic counting and calculations on a scale of 1-9 (with 9 being most proficient). This is not a standardised assessment, however it is conducted by a trained professional (Early Years Practitioner) and moderated by the local authority.
	EYFSP personal, social and emotional development	EYFSP sub scale: assesses a child's personal, social and emotional development on a scale of 1-9 (with 9 being most proficient). This is not a standardised assessment, however it is conducted by a

For peer review only - http://bmjopen.bmj.com/site/about/guidelines.xhtml

	trained professional (Early Years Practitioner) and moderated by the local authority.
EYFSP communication, language and literacy	EYFSP sub scale: assesses a child's communication, language and literacy performance on a scale of 1-9 (with 9 being most proficient). This is not a standardised assessment, however it is conducted by a trained professional (Early Years Practitioner) and moderated by the local authority.
EYFSP physical development	EYFSP sub scale: assesses a child's physical development on a scale of 1-9 (with 9 being most proficient). This is not a standardised assessment, however it is conducted by a trained professional (Early Years Practitioner) and moderated by the local authority.
EYFSP creative development	EYFSP sub scale: assesses a child's creative development on a scale of 1-9 (with 9 being most proficient). This is not a standardised assessment, however it is conducted by a trained professional (Early Years Practitioner) and moderated by the local authority.
KS1 Maths	National Standardised Test: Mathematics assessment score.
KS1 Writing	National Standardised Test: Writing assessment score.
KS1 Reading	National Standardised Test: Reading assessment score.
KS1 Science	National Standardised Test: Science assessment score.

Note: Educational features are drawn from the National Pupil Database (NPD). The NPD is a longitudinal database which holds individual-level records for all children and young people enrolled in state school, or taking national exams, and are currently residing in England. The database is large, with over 15 million records added since it's introduction (2002). The NPD holds information on a child's attainment, attendance, school context, and level of need (in addition to demographic variables). Data is provided on a quarterly basis by all state funded primary and secondary schools across England, via a dedicated submission system to the government's Department for Education.

For peer review only

Table S3: Educational feature time points

		EYFSP	KS1
Demographic features	Gender (Male)	✓	✓
	Age	✓	✓
	Summer Birth	✓	✓
	Ethnicity	✓	✓
	Deprivation Indicator (IDACI)	✓	✓
	Academic Year		✓
Additional need indicators	eFSM	✓	✓
	Mobility (KS1)		✓
	Mobility (ever)		✓
	Child Looked After Status	✓	✓
	English first language	✓	✓
Special Educational Need indicators	No SEN	✓	✓
	SEN Statement	✓	✓
	SEN School Action	✓	✓
	SEN School Action+	✓	✓
	SEN: Autism spectrum disorders	✓	✓
	SEN: specific learning difficulty	✓	✓
	SEN: moderate learning difficulty	✓	✓
	SEN: severe learning difficulties	✓	✓
	SEN: behavioural, emotional and social difficulty	✓	✓
	SEN: speech, language & communication needs	✓	✓
	SEN: hearing impairment	✓	✓
	SEN: vision impairment	✓	✓
	SEN: multi-sensory impairment	✓	✓
	SEN: physical	✓	✓
	SEN: other	✓	✓
	Mainstream education indicator		✓
School attendance and exclusion features	Attendance (%)	✓	✓
	Excluded (temporary)		✓
	Excluded (permanent)		✓
School attainment features	EYFSP knowledge of the world	✓	
	EYFSP problem solving, reasoning and numeracy	✓	
	EYFSP personal, social and emotional development	✓	
	EYFSP communication, language and literacy	✓	
	EYFSP physical development	✓	
	EYFSP creative development	✓	
	KS1 Maths		✓
	KS1 Writing		✓
	KS1 Reading		✓
	KS1 Science		✓

For peer review only - http://bmjopen.bmj.com/site/about/guidelines.xhtml

Table S4: Tuned hyperparameters

Logistic Regression	solver
Random Forest	n_estimators, max_depth, max_features, bootstrap, criterion, min_samples_split
SVM	C, cache_size, degree, gamma, kernel, max_iter, tol=0.001
Gaussian Bayes	No tuning
MLP	weight_decay, batch_size, dropout_rate, number of hidden layers

For peer review only

For peer review only - http://bmjopen.bmj.com/site/about/guidelines.xhtml

Table S5: AUC comparison between ROC and PRC analyses for population and clinical classification of ADHD.

	Population cohort		Clinical cohort	
**Classification Model**	ROC[c] AUC[d]	PRC[e] AUC[d]	ROC[c] AUC[d]	PRC[e] AUC[d]
**LR[a]**	0.900	0.101	0.694	0.244
**RF[b]**	0.86	0.070	0.688	0.257
**Random**	0.50	0.029	0.50	0.21

[a] Logistic Regression (LR), [b]Random Forest (RF), [c] Receiver Operating Characteristic curve (ROC)
[d] Area Under Curve (AUC) [e] Precision-recall curve

For peer review only

For peer review only - http://bmjopen.bmj.com/site/about/guidelines.xhtml

Table S6: Pupil characteristics and feature contribution (unweighted) for ADHD prediction in the general population sample.

Characteristics	Logistic Regression (***B*** co-efficient)	Random Forest (feature importance)
Male Gender	0.608463661	0.141773129
Summer Born (May-August)	0.109707469	0.000807359
White Ethnic group	0.201065669	0.006387057
Black Ethnic group	-0.098073916	0.000345206
Asian Ethnic Group	-0.15535868	0.011452877
Chinese Ethnic group	-0.469369952	0
Mixed Ethnic group	0.165278809	0.001506941
Other Ethnic Group	-0.065271609	0.002849315
English as first language	0.696034466	0.031602634
EYFSP: FSM eligible	0.110110336	0.001856049
EYFSP: Looked After Child	-0.058157067	0
EYFSP: IDACI score	-0.245109352	0.012435495
EYFSP: Communication, Language and Literacy	0.180721245	0.01633496
EYFSP: Knowledge of the World z score	0.436711733	0.011483452
EYFSP: Person, social, emotion z score	-0.84127724	0.047292232
EYFSP: Physical Development	-0.027708196	0.007938476
EYFSP: Creative Development	0.123449383	0.01211572
EYFSP: Problem solving, reasoning and numeracy	0.224475588	0.019006476
school mobility (ever)	-0.066303394	0.001939589
EYFSP: attendance (%)	-0.982714854	0.050421392
EYSFSP local school	-0.077583539	0.0017516
EYSFSP school type	-0.074490398	0.003640833
EYFSP: No SEN	-0.105148801	0.093914996
EYFSP: SEN School Action	0.048150906	0.008735734
EYFSP: SEN School Action Plus	0.122221511	0.030373947
EYFSP: SEN Statement	-0.044818697	0
EYFSP: SEN speech, language & communication needs	-0.113501845	0.001918025
EYFSP: SEN Autism	-0.027233555	0
EYFSP: SEN behavioural, emotional and social difficulty	-0.087858945	0.005794567
EYFSP: SEN Hearing	0.002234986	0.002198098
EYFSP: SEN Moderate learning difficulty	-0.002195983	0.000564267
EYFSP: SEN Other	0.046557778	0.00206679
EYFSP: SEN Physical	-0.043438673	0.003612997
EYFSP: SEN Specific learning difficulty	0.008096732	0
EYFSP: SEN Severe learning difficulty	-0.087701227	0.000828542
EYFSP: SEN Vision	-0.014857814	0
EYFSP: SEN Multi-sensory impairment	-0.137831643	0
KS1: Age at start	0.100899579	0.018690101
KS1 : Academic Year (e.g. 2008/9)	-0.046794005	0.022486708
KS1: mainstream school	-0.064770905	0.002768431

For peer review only - http://bmjopen.bmj.com/site/about/guidelines.xhtml

KS1: FSM eligible	0.113473204	0.001361182
KS1: IDACI score	0.17501001	0.006385568
KS1: Looked After Child	0.054056081	0.001767296
KS1: Maths score	-0.164521477	0.032731843
KS1: Writing point score	-0.761201837	0.10916803
KS1: Read Score	0.001961117	0.035857909
KS1: Science score	-0.028166159	0.010063037
KS1 school mobility	0.054731598	0.001614064
KS1: attendance (%)	0.957564403	0.059408817
KS1: Excluded	0.125124512	0.004965946
KS1: Exclusion	0.024925737	0.005752298
KS1: Not SEN	-0.198059867	0.08701934
KS1: SEN School Action	0.063043447	0.003333059
KS1: SEN status: school action plus	0.175904692	0.020499615
KS1: SEN: speech, language & communication needs	-0.030230103	0.000800672
KS1: SEN Autism	0.147169063	0.002138406
KS1: SEN behavioural, emotional and social difficulty	-0.087858945	0.00538704
KS1: SEN Hearing	0.002234986	0.001026292
KS1: SEN Moderate learning difficulty	-0.002195983	0.000752458
KS1: SEN Multi-sensory impairment	-0.137831643	0
KS1: SEN Other	0.046557778	0.007853115
KS1: SEN Physical	-0.043438673	0.004452396
KS1: SEN Specific learning difficulty	0.008096732	0.000377725
KS1: SEN Severe Learning Difficulty	0.043312749	0
KS1: SEN Statement	0.09702835	0
KS1: SEN Vision	-0.014857814	0

For peer review only - http://bmjopen.bmj.com/site/about/guidelines.xhtml

Figure S1: Pre-processing fairness pipeline

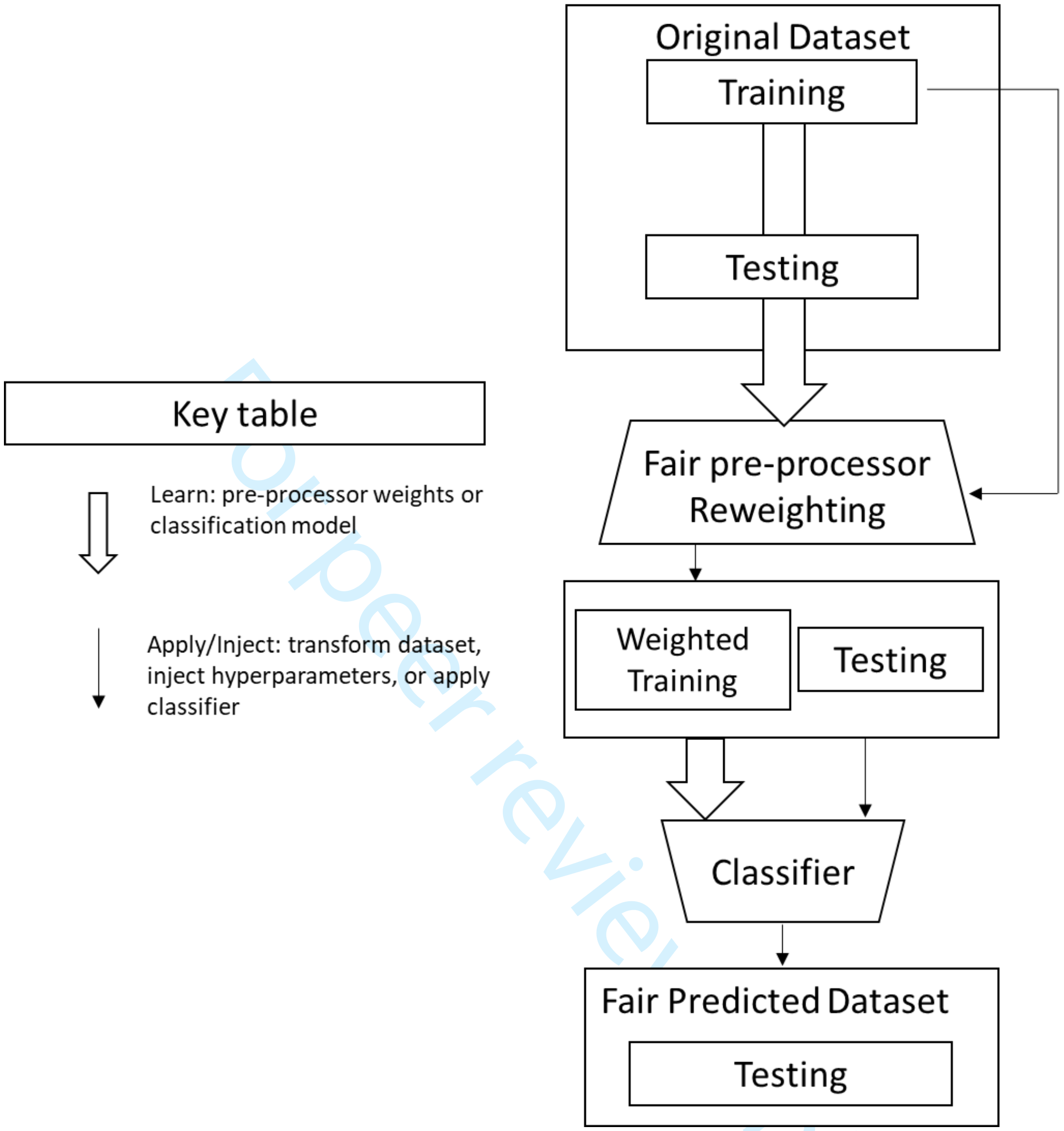

For peer review only - http://bmjopen.bmj.com/site/about/guidelines.xhtml

Figure S2: Precision-Recall curves comparing Logistic Regression(LR), Random Forest (RF) and a Random classifier for ADHD diagnosis within a population cohort

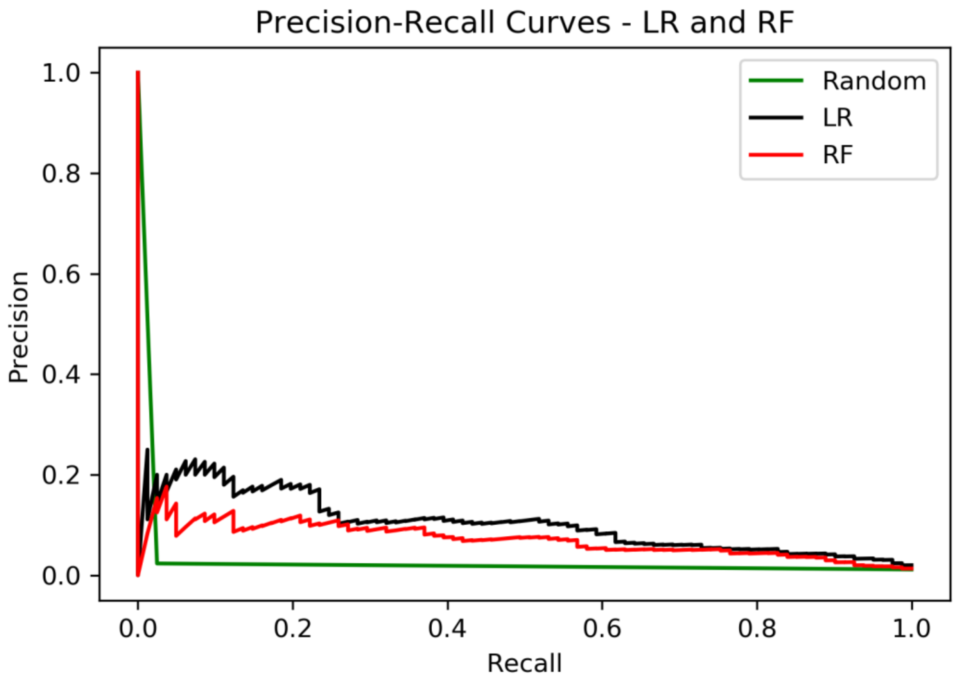

Figure S3: Precision-Recall curves comparing Logistic Regression(LR), Random Forest(RF) and a Random classifier for ADHD diagnosis within a clinical cohort

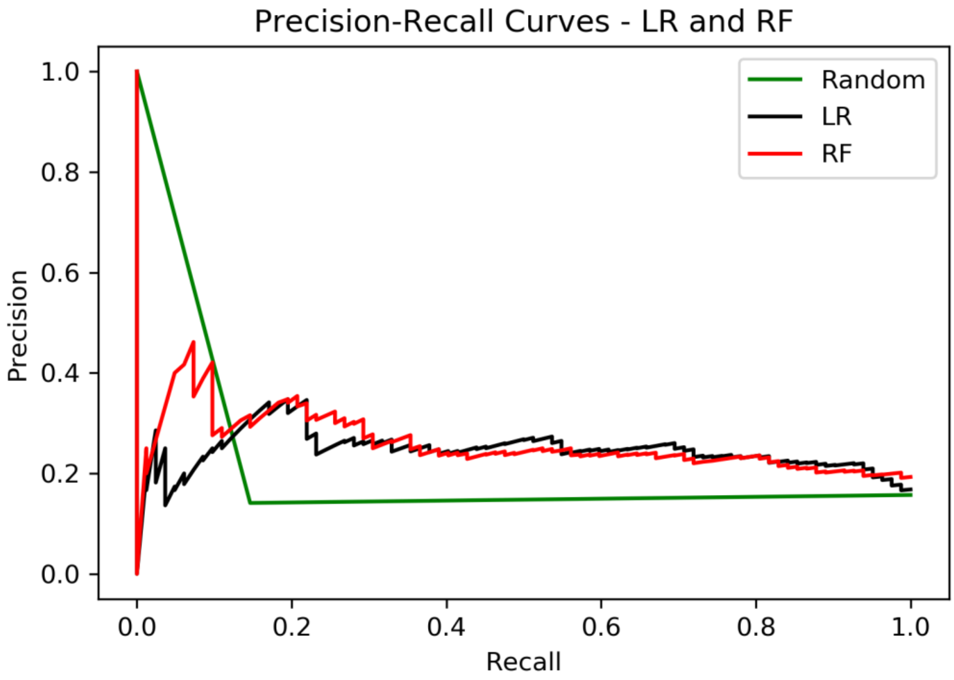

For peer review only - http://bmjopen.bmj.com/site/about/guidelines.xhtml
